# Systematic literature review on impacts of COVID-19 pandemic and corresponding measures on mobility

**DOI:** 10.1007/s11116-023-10392-2

**Published:** 2023-04-25

**Authors:** Kwang-Sub Lee, Jin Ki Eom

**Affiliations:** Railroad Policy Research Department, Korea Railroad Research Institute, 176 Railroad Museum Road, Uiwang-Si, 16105 Gyeonggi-Do Korea

**Keywords:** COVID-19, Measures, Social distancing, Mobility, Travel behavior

## Abstract

The unprecedented COVID-19 outbreak has significantly influenced our daily life, and COVID-19’s spread is inevitably associated with human mobility. Given the pandemic’s severity and extent of spread, a timely and comprehensive synthesis of the current state of research is needed to understand the pandemic’s impact on human mobility and corresponding government measures. This study examined the relevant literature published to the present (March 2023), identified research trends, and conducted a systematic review of evidence regarding transport’s response to COVID-19. We identified key research agendas and synthesized the results, examining: (1) mobility changes by transport modes analyzed regardless of government policy implementation, using empirical data and survey data; (2) the effect of diverse government interventions to reduce mobility and limit COVID-19 spread, and controversial issues on travel restriction policy effects; and (3) future research issues. The findings showed a strong relationship between the pandemic and mobility, with significant impacts on decreased overall mobility, a remarkable drop in transit ridership, changes in travel behavior, and improved traffic safety. Government implemented various non-pharmaceutical countermeasures, such as city lockdowns, travel restrictions, and social distancing. Many studies showed such interventions were effective. However, some researchers reported inconsistent outcomes. This review provides urban and transport planners with valuable insights to facilitate better preparation for future health emergencies that affect transportation.

**Supplementary Information:**

The online version contains supplementary material available at 10.1007/s11116-023-10392-2.

## Introduction

The novel coronavirus outbreak (COVID-19), was first reported from Wuhan, China on December 31, 2019 (Gkiotsalitis and Cats, 2021; De Vos 2020). The World Health Organization (WHO) declared COVID-19 a global pandemic on March 11, 2020, because the highly contagious disease had rapidly spread, affecting people’s lives worldwide (Mashrur et al. 2022; Zhang et al. 2020b; De Haas et al. 2020; Hadjidemetriou et al. 2020). The current crisis differs from previous epidemic trends (i.e., SARS or H1N1) in that it is global, difficult to contain, has a rapid spread rate, and a high death toll (Borkowski et al. 2021). Given the pandemic’s severity, it is a crucial that governments control the spread. Therefore, they implemented a combination of measures, applying various approaches to isolate outbreaks and avoid further exposures by reducing close contact with the virus (Jaekel and Muley 2022; Wang et al. 2022; Arimura et al. 2020; Lau et al. 2020). These countermeasures included forced or recommended measures, such as city lockdowns, confinement, halting domestic and international flights, travel restrictions, workplace closures, and social distancing (Chen et al. 2022a; Lu et al. 2021; Pan et al. 2020; Chinazzi et al. 2020; Shakibaei et al. 2020). However, the pandemic is still not fully under control and its impacts persist as of March 2023, including a huge death toll and negative influences on quality of life, such as economic losses, business closures, and social activities (Kim 2021; Tan and Ma 2021).

According to WHO, COVID-19 is respiratory and spreads mainly through contact with an infected person (WHO, 2021; Moslem et al. 2020). Spread is inevitably associated with human movement, and the transport sector plays an important role in reducing the spread of infection (Rothengatter et al. 2021; Liu et al., 2020a; Moslem et al. 2020; Sokadjo and Atchade 2020; Lee et al. 2020a; Muley et al. 2020). Because there is a strong correlation between infectious diseases and movement of people, many researchers argued that travel restrictions could contribute to limiting the virus (Chen et al. 2020, 2022a; Choi et al. 2022; Zhang et al. 2021b; Fatmi 2020; Liu et al. 2020b; Muley et al. 2020). For instance, several studies showed that population emigration from Wuhan was highly correlated to imported cases in other Chinese cities Su et al. 2022a; Liu et al. 2020b; Chen et al. 2020; Zhao et al., 2020a; Shi and Fang 2020; Liu et al., 2020a), and that lockdown policies effectively slow COVID-19 spread (Gramsch et al. 2022; Mars et al. 2022; Chen et al. 2022a; Li et al. 2021a; Wen et al. 2021; Anzai et al. 2020; Aloi et al. 2020; Cintia et al. 2020; De Haas et al. 2020).

Given the high transmissibility, limited epidemiological understanding, and lack of a specific COVID-19 treatment, understanding human mobility and containment measure effects is crucial to mitigating COVID-19’s impact (Gramsch et al. 2022; Liu et al. 2022; Ciuffini et al. 2021; Hadjidemetriou et al. 2020; Cintia et al. 2020; Muley et al. 2020) reviewed previous transport and infectious disease literature, including COVID-19, and found that the transport sector has a two-fold role during an infectious disease outbreak: controlling infection spread and assessing the impact of reduced outdoor activities on the transport sector. With different countries’ rapidly changing environments, it is extremely difficult to quantify the magnitude of mobility related measures’ impact and draw a general and consistent conclusion (Tan and Ma 2021). Although vaccine is now available, some moderate regulations, such as social distancing and personal protective measures, might remain for a long time to mitigate the pandemic and to prepare for another pandemic wave. Given that the COVID-19 pandemic differed from previous epidemic trends, COVID-19 research may not be directly applicable to future epidemics. However, understanding changes to travel behavior characteristics during COVID-19, and examining factors affecting travel patterns and various preventive measures’ effectiveness, provides important information for policy makers.

A few literature reviews have been published so far, but their topics are limited to a specific transportation field (e.g., the impacts of COVID-19 on public transport by Gkiotsalitis and Cats, 2021) and a specific subject (e.g., transportation policies and mitigation strategies by Peralvo et al. 2022; and the built environment and human factors by Alidadi and Sharifi 2022), or to review in a particular way (e.g., bibliometric analysis by Benita 2021). There is one study similar to our intention that Muley et al. (2020) systematically discussed the impact of COVID-19 on the transport sector. However, they did not consider studies on the effects of various government policies in response to COVID-19. In particular, they reviewed the subjects of studies up to June 2020, and since many COVID-19-related papers are constantly being published, it is necessary to update on the latest research. Accordingly, our study’s key objective is to synthesize evidence from the scientific literature and case studies (published up to March 2023) on the impact of COVID-19 on urban transportation, to assist policy makers and urban and transportation planners better prepare for future health emergencies. We review studies on COVID-19’s impact on human mobility and the corresponding governments’ countermeasures to present a comprehensive synthesis of previous studies with diverse perspectives, and to discuss future research needs. We conduct thorough literature reviews, identify and classify the material by subject, and present key results and controversial issues.

The specific topics covered in this study and the structure of the paper are as follows. Chapter 2 describes the methodology (e.g., literature review strategy and selection criteria) of this study. Chapters 3 and 4 review studies on COVID-19 and government measures and their impact on the transport sector. In fact, changes in travel patterns related to the pandemic may have various causes, such as government measures to limit the spread of the virus, people’s compliance with such measures, and changes in activities and travel behavior that each person has selectively taken to avoid contagion. However, it seems difficult to distinguish the exact cause and effect. Accordingly, we distinguish between studies that do not analyze the effectiveness of government policies (Chap. 3) and studies that explicitly analyze the effectiveness (Chap. 4). In Chap. 3, literatures on the overall impacts of the COVID-19 outbreak on mobility regardless of the presence of the government’s specific measures are also divided into: (1) studies on mobility changes quantitatively analyzed based on observed transportation data including mobile phone data and Google Mobility reports, and (2) survey-based studies to investigate changes in personal travel behavior according to demographic and socioeconomic characteristics. The first topic is further subdivided by the relationship between COVID-19 and human mobility (Sect. 3.1) and impacts on overall mobility (Sect. 3.2), public transportation (Sect. 3.3), and other impacts (Sect. 3.4). Section 3.5 focuses on changes in personal travel and activity behavior based on surveys. Chapter 4 reviews studies that explicitly analyzed how the government’s specific measures to contain the spread of COVID-19 affect mobility and whether reduced traffic effectively reduces the spread of infection. Specifically, we review studies on travel restriction policies’ impacts on reducing human mobility (Sect. 4.1), the relationship between travel restriction policy and COVID-19 transmission (Sect. 4.2), and conflicting findings and issues on travel restriction policy effects (Sect. 4.3). Chapter 5 discusses what future research topics are needed and then concludes in Chap. 6.

## Methodology

### Literature review strategy

Given the unprecedented severity of COVID-19 and the extent of spread, many studies have been published within a short time frame. The review was conducted through five steps (Snyder 2019; Wolfswinkel et al., 2013; Khan et al. 2003): (1) developing research questions; (2) searching for relevant studies based on inclusion and exclusion criteria; (3) assessing studies’ quality to identify literature relevant to our research interests; (4) identifying research topics and classifying them by the subject; and (5) summarizing and synthesizing the selected studies’ results.

### Literature selection: inclusion and exclusion criteria

After the systematic review objective was defined, we conducted a literature search using search engines, including Google Scholar and three of the most recognized academic databases that cover top-notch journals, Scopus, Science Direct, and Web of Science. The search was restricted to journal articles that included selected keywords. We used comprehensive combinations of search terms; additionally, two main search strings were employed and combined using parentheses with “AND,” one specifying all the relevant keywords for “COVID-19” and “transport,” the other specifying keywords such as “mobility,” “impact,” “effect,” “travel behavior,” “restriction,” and “measurement.”

After relevant literature was initially identified based on titles, abstracts, and conclusions, we applied the following inclusion criteria to narrow the results: (1) studies published from 2020 to the present (March 2023); (2) studies with access to the full text written in English; (3) all review articles, empirical studies, conferences or proceedings, peer-reviewed journals, including quantitative or qualitative case studies; and (4) studies examining all types of human mobility, including private car, public transport (bus, railway), bicycle, and personal mobility. However, although impacts related to the aviation sector were not specifically excluded, the analysis focused on intra-country mobility as much as possible, because inter-country movement is directly affected by each country’s immigration policy. From the selected studies, we screened article relevance using the following exclusion criteria: (1) studies covering COVID-19 in general and not related to the transport sector (i.e., COVID-19 impacts not related to human mobility and activity); (2) studies that focused on personal health, pharmacological intervention, epidemiological or pathological evidence; and (3) studies on freight/marine/water transport.

### Literature extraction

Before the full text assessment, we also reviewed reference lists for relevant literature and discovered additional relevant articles through forward and backward reference tracing, adding them to the search lists to complement the literature identified through database searches. Subsequently, duplicates were removed, and the remaining studies were further screened for relevance and scope by examining each article’s abstract, introduction, and conclusion. After filtering, 364 articles remained for the final analysis. We thoroughly reviewed each study’s content and conducted thematic analyses to categorize studies based on their topics and study perspectives. This approach effectively identified each study’s purpose, data, and results, and grouped them into major topics and sub-topics. When the study subject and transport means were similar across multiple studies, those not significantly meaningful to this study’s review subject were not included in the analysis. The articles relevant to each subject were extracted and summarized in tables that included publication details (author(s) and year), study area, research objective and method, data type, and transport mode type.

## Overall impacts of the pandemic on mobility regardless of Government’s measures

This chapter reviews the literatures on the overall impacts of the COVID-19 outbreak on mobility regardless of the presence or absence of the government’s specific measures. Specifically, existing studies are discussed under the following topics: (1) the relationship between human mobility and COVID-19 transmission; (2) the impact of COVID-19 on overall mobility based on observed data; (3) the impact on public transportation; (4) other impacts; and (5) changes in personal travel behavior based on survey data.

### Relationship between human mobility and COVID-19 transmission

Increased traffic volume increases the possibility of contact between people and thus increases potential COVID-19 spread. Extensive research investigated the link between human mobility and COVID-19 spread, and showed a positive correlation (Table 1). Because COVID-19 was first detected in Wuhan, China, many studies focused on data from China Shi and Fang 2020; Chen et al. 2020; Liu et al. 2020b; Zhao et al., 2020a). For example, Shi and Fang (2020) investigated the temporal relationship between daily outbound traffic from Wuhan to 31 Chinese provinces and COVID-19 incidence during the virus’s early spread in 2020. Outbound traffic volume was positively associated with COVID-19 incidence in all provinces, with correlation coefficients ranging from 0.22 to 0.78, and statistically significant at the 95% level. Chen et al. (2020); Liu et al. (2020b) found that correlation coefficients between population emigration from Wuhan ranged from 0.597 to 0.996 depending on regions and mobility patterns. Moreover, a significant and positive association was observed between public transportation daily frequency—including flights, buses, and trains operating from Wuhan—and the number of COVID-19 cases (Zheng et al. 2020).

Case studies outside China showed similar patterns (Jaekel and Muley 2022; Yang et al. 2021; Cintia et al. 2020). Mobility patterns derived from mobile phone data in 25 US counties showed a strong correlation, with Pearson correlation coefficients above 0.7 for 20 of the 25 counties (Badr et al. 2020; Kissler et al. 2020) observed that the mean estimated prevalence of COVID-19 infection by borough in New York City was strongly negatively correlated with reduced commuting (-0.88Iacus et al. (2020b) also confirmed that human mobility (internal and outbound movements) had a high impact on initial virus spread in case studies of France, Italy, and Spain, with between 52% and 92% in France, up to 91% in Italy, and up to 75% in Spain. Kartal et al. (2021) revealed a cointegrated relationship between mobility and pandemic indicators through the Toda-Yamamoto causality test.

There are studies examining COVID-19’s impact on each type of transport mode to evaluate the contribution of different transport modes to virus spread. Several studies found a strong correlation between air traffic (e.g., airline passenger, number of airports, and flight routes) and COVID-19 spread (Su et al. 2022a, b; Lau et al. 2020; Sokadjo and Atchade 2020; Oztig and Askin 2020) found that both domestic and international passenger volumes in China were strongly associated with domestic and international COVID-19 cases. The results indicated that adequate measures are necessary to prevent a long-term crisis, such as on-site disease detection, temporary passenger quarantine, and limited air traffic operation. Oztig and Askin (2020) employed a negative binomial regression analysis on data from 144 countries, including population density as a control variable, and found a positive association between high numbers of airports in a country and high numbers of infected patients. A strong and significant association was also found between travel volume by train and the number of confirmed COVID-19 cases (Pang et al. 2023; Zhao et al. 2020b) found that one more high-speed railway (HSR) train originating from Wuhan each day increases the cumulative number of COVID-19 cases in a city by about 10%. Zhao et al. (2020b) estimated that a 10% increase in the number of train passengers from Wuhan to major cities in China resulted in an 8.27% increase in infections. However, cars and flights were not statistically significant in the study. Conversely, Zhang et al. (2020b) presented slightly different results. Flight and high-speed train frequencies in and out of Wuhan, China were positively and significantly associated with number of confirmed cases in the destination cities at the level of 1% and 10%, respectively. In contrast, coach (inter-city bus) services were not significantly associated with imported confirmed cases, presumably because most of coach travelers use the service for relatively short trips. Therefore, the authors argued that limiting air transport from a pandemic center is the first measure to employ to reduce travel related imported infections. Another study suggests that accessibility is related to the spread of COVID-19. Carteni et al. (2021) focused on the hypothesis that areas with higher accessibility were more easily reached by the virus. Based on data from Italy, the regression model showed that transport accessibility, population, population density, and particulate matter (PM), were significantly related to COVID-19 cases. Rail-based transport accessibility (39.7% in weight) was the best predictor for number of COVID-19 infections, followed by population and population density (about 14%), and territorial and pollutant variables (9.3%).

**Table 1:** Overview of key studies on relationship between human mobility and COVID-19 transmission

Ref	Study area	Method	Data used	Mode	Key findings
Badr et al. (2020)	USA (25 counties)	Generalized linear model	O/D trip matrices (cell phone data), COVID-19 data	Overall mobility	Mobility patterns are strongly correlated with decreased COVID-19 case growth rates (correlation coefficients above 0.7 for 20 of the 25 counties).
Carteni et al. (2021)	Italy	Regression	COVID-19 reports, rail service characteristics, survey	Overall mobility	Transport accessibility is the variable that better explained the number of Covid-19 infections (about 40% in weight).
Chen et al. (2020)	China (Wuhan & Hubei)	Bayesian space-time model	COVID-19 cases, population migration data	Overall mobility	The case numbers of different provinces and cities of Hubei province were highly correlated with the emigrated populations from Wuhan.
Cintia et al. (2020)	Italy	Regression	Daily mobility flows (mobile phone data), COVID-19 related data	Overall mobility	A strong relationship between the mobility flows and the net reproduction number during the lockdown.
Habib et al. (2021)	10 countries	Non-linear modeling	COVID-19 & mobility data	Overall mobility	A significant positive association between COVID-19 and transportation mobility in the USA, UK, Spain, Italy, Canada, France, Germany & Belgium.
Iacus et al. (2020b)	France, Italy, & Spain	Regression	O/D trip (mobile phone), number of deaths by COVID-19, distance	Overall mobility	Mobility alone can explain up to 92% of the initial spread in France and Italy.
Kartal et al. (2021)	Turkey	Toda-Yamamoto causality test	COVID-19 & mobility data	Overall mobility	There is cointegration between the variables in the long term, and is an econometric causality between mobility indicators and pandemic indicators.
Kissler et al. (2020)	USA (New York)	Bayesian distribution analysis	Trip data (Facebook data), COVID-19 test results	Overall mobility	COVID-19 infection was lowest in boroughs with the greatest reductions in morning movements out of and evening movements into the borough.
Lau et al. (2020)	China & international	Regression	Air passenger volume; flight routes; COVID-19 cases	Air	A strong correlation between COVID-19 cases and passenger volume.
Lee et al. (2020a)	Korea	Regression	Daily national traffic data, COVID-19 cases	Overall mobility	All regions except Incheon showed negative linear relationships between numbers of newly confirmed cases and traffic.
Liu et al. (2020b)	China (350 cities)	Regression model, simulation	COVID-19 confirmed cases, mobility data	Overall mobility	All mobility patterns correlated with the spread of the virus, while the correlations dropped with the implementation of travel restrictions.
Oztig & Askin (2020)	144 countries	Negative binomial regression	Airline passengers & airports, population density, elderly people	Air	A positive relationship between volume of airline passenger traffic and numbers of COVID-19 patients.
Pang et al., (2023)	China (Wuhan & others)	Gravity model	HSR train operations, COVID-19 cases, population	HSR	One more HSR train originating from Wuhan each day before the Wuhan lockdown increases the cumulative number of Covid-19 cases in a city by about 10%
Shi & Fang (2020)	China (31 provinces)	ARIMA model	COVID-19 cases, traffic volume (cellphone location), distance, GDP	Overall mobility	The volume of outbound traffic from Wuhan was positively associated with COVID-19 incidence in all provinces, with correlation coefficients between 0.22–0.78 (all P < 0.05).
Sokadjo & Atchade (2020)	World	Various regression	COVID-19 case & passenger air traffic data	Air	When passenger air traffic increases by one unit, the number of cases increases by one new infection.
Sy et al. (2020)	USA (New York)	ANOVA, regression	COVID-19 case, subway ridership, demographic & socioeconomic data	Subway	Increased mobility & most of sociodemographic variables were associated with a higher rate of COVID-19 cases per 100k.
Yuksel et al. (2020)	Canada	Regression	Mobility (Apple, Google, Facebook), weather, COVID-19 data	Overall mobility	The degree of social distancing under strict restrictions is bound by choice.
Zhang et al. (2020b)	China (Wuhan and other cities)	Gravity model	COVID-19 cases, transport service, GDP, distance & location of cities, airport/HSR station	HSR, coach & air	Frequencies of air flights and HST services out of Wuhan are significantly associated with the number of COVID-19 cases in the destination cities.
Zhao et al. (2020a)	China (10 city clusters)	Regression	Passengers (cellphone location), COVID-19 cases data	Overall mobility	A strong and significant association between travel by train and the number of COVID-19 cases.
Zhao et al. (2020b)	China (Wuhan & 6 cities)	Regression	Passengers by car, train & flight; COVID-19 confirmed cases data	Car, train & air	A statistically significant positive association between the load of passengers multiplied by the local infectivity in Wuhan and the number of cases reported outside Wuhan.
Zheng et al. (2020)	China	Pearson correlation analysis	Frequencies of public transport, distance, COVID-19 cases	Flight, bus & train	A positive association between the frequency of flights, trains, & buses from Wuhan and the daily as well as the cumulative numbers of COVID-19 cases in other cities.

### Impact of COVID-19 on overall mobility

Because of the COVID-19 pandemic and government countermeasures, all cities worldwide experienced reduced traffic volumes, which may have resulted from various causes, such as deceased voluntary outside activities owing to fear of COVID-19 infection, and/or government orders (i.e., travel restriction, social distancing policies) implemented to mitigate spread.

A range of studies examined the impact of COVID-19 on mobility using different data sources and research perspectives (Table 2). The impact of COVID-19 on transportation demand was greatest in the early stages of the outbreak, and early studies focused on this. Gonzalez et al. (2021) found that public and private mobilities at the peak of the pandemic dropped to 95% and 86% of pre-COVID-19 levels in Spain. C2SMART (Connected Cities with Smart Transportation Center) releases monthly reports on mobility changes in New York and Seattle, US as case studies to analyze transit ridership, bridge and tunnel traffic, travel time, and number of crashes during the pandemic (Gao et al. 2020a, d; Bernardes et al. 2020). After the stay-at-home order was implemented in New York, both transit ridership and general traffic volume dropped, with transit ridership severely impacted, dropping 94% in the peak period as of March 23, 2020 (Gao et al. 2020a) compared to the 2019 statistics. It remained down at 91% in April (Gao et al. 2020d), improving to 80% in the first week of July (Bernardes et al. 2020). Reduced traffic volumes owing to the stay-at-home policy resulted in a decrease of average travel times as well: dropped by 38% during the third week of February (Gao et al. 2020a). In contrast, cycling increased by 55% in a temporary mode shift, and all traffic safety indicators improved (vehicle collisions dropped up to 77%, pedestrian injury/fatality decreased 51%, and cyclist injury/fatality in crashes decreased 31%) (Gao et al. 2020a). A US city, Seattle, experienced similar COVID-19 mobility impacts (Gao et al. 2020d). Highway traffic volume in the US state, Florida, also decreased by 47.5%, compared to the 2019 statistics (Parr et al. 2020). Korea’s average daily traffic volume in early 2020 also differed substantially from the 2019 volume, decreasing from 149 million vehicles in 2019 to about 144 million vehicles in 2020, a 9.7% decrease (Lee et al. 2020a). Canada’s mobility trends showed a clear, large reduction in mobility to non-residential locations after the state of emergency was declared (Chen, 2020). COVID-19 also significantly reduced taxi trips, and affected taxi trips’ travel speed (increased by 29.4%), travel time (decreased by 22.6%), and average distance (increased by 2.4%) (Nian et al. 2020). Average daily taxi trips in February 2020 were only 11.3% of those in May 2019. Nighttime taxi trips (9 PM – 5 AM) were significantly impacted dropping to 8.5% of the normal period. The impact of COVID-19 was greatest at the beginning of the epidemic, and the next waves of the pandemic seem to be less than the initial ones (Pozo et al. 2022; Advani et al. 2021; Konecny et al. 2021; Rasca et al. 2021). For example, subway traffic in 2020 in the UK fell to 5% during the first lockdown (from April to July), recovered to 37% before the second lockdown, and then fell back to 25% during the second lockdown in November (Vickerman 2021).

**Table 2:** Overview of key studies on impact of COVID-19 on overall mobility

Ref	Study area	Method	Data used	Mode	Key findings
Abu-Rayash & Dincer (2020)	Selected cities in the world	Mathematical model	Transportation related, Mobility Index data	Overall mobility	As of the end of June 2020, cities with higher than 50% mobility index include Brussels, Singapore, Stockholm, Lyon, Paris, Moscow, and Hong Kong with the highest mobility index of 76%.
Arimura et al. (2020)	Japan (Sapporo)	Comparative analysis	Mobile spatial statistics	Overall mobility	The city’s residents have been more likely to stay home and less likely to travel to the center area, resulting in a decrease of up to 90% of the population density in crowded areas.
Bucsky (2020)	Hungary (Budapest)	Comparative analysis	Daily transport volume & ridership data	All types	Public transport experienced the greatest reduction in demand (80%), while cycling (23%) and bike sharing (2%) saw the lowest decrease.
Chen (2020)	Canada	Regression	Google reports, COVID-19 cases & deaths data, temperature	Overall mobility	Visits to nonresidential locations sharply dropped, with a corresponding increase in visits to residential locations.
Cui et al. (2020)	USA (Seattle)	Comparative analysis	Loop detector data, public agency data, COVID-19 datasets	Overall mobility	Developed Traffic Performance Score (TPS) that incorporates multiple parameters for measuring network-wide traffic performance.
Gao et al. (2020a)	USA (New York)	Comparative analysis	Ridership, weigh-in-motion, NYC open data	Overall mobility	Ridership data show steep declines in both transit ridership and vehicular traffic after the stay-at-home order
Gao et al. (2020d)	USA (NY, Seattle)	Comparative analysis	Ridership, weigh-in-motion, NYC open data	All types	Low volume in transit ridership and motor vehicle trips: Subway ridership remained down 91% and vehicular traffic via MTA bridges and tunnels was down 68% in April 2020 vs. 2019 in NY.
Gonzalez et al. (2021)	Spain	Comparative analysis	Smart card, bluetooth traffic monitoring data	Car, public transit	Public and private mobility dramatically decreased to 95% and 86% of their pre-COVID-19 values.
Hasselwander et al. (2021)	Philippines	Analytical analysis	Cell phone & GPS data	All types	Travelers most reliant on public transport were disproportionately affected by lockdowns.
Jia et al. (2020)	China (Wuhan & other cities)	Various models	Mobile phone data, COVID-19 case, population & GDP	Overall mobility	The distribution of population outflow from Wuhan accurately predicts the relative frequency and geographical distribution of infections with COVID-19.
Kaufman et al. (2020)	USA (New York)	Comparative analysis	Public data	All types	On April 12th, 2020, subway ridership had dropped 96%. Commuter rail suffered the greatest losses at up to 97.9% less than 2019 levels; followed by subways at 91.7%. and buses at 78.3%.
Lee et al. (2020b)	USA	Comparative analysis	Mobile device location, COVID-19 case, population data	Overall mobility	Public movements were decreased after the national emergency declaration. The population staying home has increased in all states.
Lee et al. (2023)	Korea	Comparative analysis	Smart card, private vehicle records	Car, public transit	A significant decrease in trip frequency was found during non-peak hours on weekdays and during weekends. People reduced their daily trip distances: private vehicle usage increased for shorter trip distances while bus usage dropped regardless of the ranges of trip distances under the pandemic
Nian et al. (2020)	China (Chongqing)	Spatial lag model	Taxi trip, POI data	Taxi	The number of taxi trips dropped sharply, and the travel speed, travel time, and spatial distribution of taxi trips had been significantly influenced during the epidemic.
Parr et al. (2020)	USA (Florida)	Comparative & statistical analysis	Traffic count data	Highway traffic	Compared to similar days in 2019, overall statewide traffic volume dropped by 47.5%. There were also differences between rural and urban areas.
Ruiz-Euler et al. (2020)	USA (6 urban centers)	Comparative analysis	Mobile device location, census data	Overall mobility	A different drop rate of mobility for high & low income groups, which we call the mobility gap.
Wang et al. (2020)	USA (New York)	Agent-based simulation	Vehicle traffic, subway ridership, Apple report data	Car, subway, walk, bike	A full reopening would only see as much as 73% of pre-COVID transit ridership and an increase in the number of car trips by as much as 142% of pre-pandemic levels, assuming mode preferences held during the crisis are maintained.
Wen et al. (2021)	New Zealand	Comparative analysis	Google & Apple reports	Overall mobility	Lockdown had a significant impact on the reduction in mobility and variation in transport mode.

While previous studies used various databases, extensive literature used aggregated location data obtained from mobile phones, including Google Community Mobility reports and Apple Mobility Trends reports, to quantify COVID-19’s impact (Askitas et al. 2020; Tirachini and Cats 2020; Carteni et al. 2020; Schlosser et al. 2020; Pullano et al. 2020; Klein et al. 2020b; Gao et al. 2020b; Yabe et al. 2020; Galeazzi et al. 2021; Santamaria et al. 2020; Iacus et al. 2020b). Many researchers emphasized location data’s usefulness for modeling disease spread (Heiler et al. 2020), providing empirical evidence of human mobility (Couture et al. 2022), investigating the effects of different types of government interventions on human mobility, and monitoring the impact of such measures on the epidemic trajectory (Pepe et al. 2020). Using mobile device location data, Lee et al. (2020b) found that US nationwide mobility trends changed rapidly around March 13, when the national emergency was declared, and daily movements in general decreased; the percentage of people staying home rapidly increased from 20% on normal days (benchmark week, Feb. 3 to Feb. 16, 2020) to 35% after the outbreak (Apr. 6 to Apr. 12, 2020); out-of-county trips decreased from 28 to 23%; average trip distance dropped from 40 miles to 23 miles; and number of trips per person decreased from 3.7 to 2.7. Based on Google Mobility Report data, even comparing two countries with different characteristics, Germany and Qatar, the impact on the transport sector (e.g., correlations between traffic volume and government measures) was found to be similar (Jaekel and Muley 2022). Using smart card and private vehicle records in Korea, Lee et al. (2023) found that trip frequency was significantly decreased during non-peak hours on weekdays and during weekends. In addition, private vehicle usage increased for shorter trip distances, while bus usage dropped regardless of trip distances. Mobile phone data and Google and Apple reports were also used for other studies to find a correlation between the outflow of people and the reported COVID-19 cases with an eight-day time lag (Heiler et al. 2020), develop daily time-series’ of different mobility metrics (Pepe et al. 2020), investigate the impact of COVID-19 on changes in community mobility and variation in transport modes during COVID-19 alert levels (Wen et al. 2021), and examine changes in population density and visualize spatial population distributions (Arimura et al. 2020).

A few studies developed models or simulations to investigate the impact of COVID-19 on future mobility (Peng et al. 2023; Wang et al. 2020). Using MATSim, an agent-based simulation model, and assuming that the mode preference during the pandemic is maintained, Wang et al. (2020) predicted that a full reopening scenario of the NYC transportation system would result in 73% of pre-COVID transit ridership owing to changed mode preferences, while increasing car traffic as much as 142% of pre-pandemic levels. When limiting transit capacity to 50%, transit ridership would decrease by as much as 64% of pre-COVID ridership, while increasing the number of car trips to as much as 143% of pre-pandemic levels.

Other studies examining COVID-19’s impact on mobility focused on heterogeneous impacts on socioeconomic demographics or across space (Pan and He 2022; Habib et al. 2021; Guzman et al. 2021) found significant inequalities between income groups with respect to access to essential services in Bogota. Lee et al. (2020b) found that a higher income group was more likely to stay home after the national emergency declaration, and a higher density group tended to have lower trip distance after the outbreak. Ruiz-Euler et al. (2020) and Yang et al. (2021) also found different rates of reduced mobility owing to COVID-19 for high- and low- income groups, called the mobility gap. The second phase of the pandemic also showed heterogeneous changes in travel behavior according to individual attributes (e.g., age, gender, education level, marital status, income, etc.) (Jiao and Azimian 2021; Glaeser et al. 2022) estimated that total cases per capita decreased by 19% when mobility dropped by 10% in five US cities. The authors observed substantial heterogeneity across space and over time: east coast cities (i.e., NYC, Boston, and Philadelphia) had stronger effects than Atlanta and Chicago. For these differences, the authors presumed to reflect the initial infection rate rather than mobility characteristics.

### Impact of COVID-19 on public transportation

The previous section confirmed that all countries worldwide experienced a pandemic related mobility drop, and public transportation was one of the most disrupted sectors (Table 3). A remarkable drop in public ridership was reported from many cities worldwide, with a 93% drop in the worst affected cities (Pozo et al. 2022; Medlock et al. 2021; Hasselwander et al. 2021; Gkiotsalitis and Cats, 2021; Aloi et al. 2020; Ahangari et al. 2020). Jiang and Cai (2022) found that for each additional local COVID-19 cumulative case within 14 days, subway ridership decreased by 0.091% in Beijing and 0.112% in Shanghai. Because public transport vehicles and stations are perceived as high risk, and fear of contagion between travelers was related to higher passenger density in a limited physical space, governments in many countries implemented restriction policies to limit or discourage public transport use, and some public transport operators reduced their services (Marra et al. 2022; Kłos-Adamkiewicz and Gutowski 2022; Jenelius and Cebecauer 2020; Tirachini and Cats 2020; Gkiotsalitis and Cats, 2021). However, public transportation is one of the most important modes of mobility, because it is sustainable and transports people on a large scale. Many transit dependent riders do not have access to a private vehicle (Pawar et al. 2020; Shakibaei et al. 2020), especially low income and historically marginalized people, who experience further loss of mobility when public transport is restricted (Suman et al. 2020; Wilbur et al. 2020; Shaheen and Wong 2020).

Regardless of the public transit transmission risk controversy, when traffic volume decreases owing to COVID-19, the reduced ridership impact is much more severe than mobility changes related to private cars. Several studies focused on this issue, examining unprecedented decline in demand and revenue, limited capacity, and social equity (Pozo et al. 2022; Shelat et al. 2022; Hasselwander et al. 2021). For example, public transport ridership decreased by about 80%, while the percentage of people using a car increased from 43 to 65%, and cycling (reduced by 23%) and bike sharing (reduced by 2%) were not significantly impacted in Budapest, Hungary (Bucsky 2020). A similar trend was reported in New York City. Subway ridership dropped 96% on April 12, 2020, compared to that before the pandemic (Kaufman et al. 2020). Commuter rail use in New York (dropped up to 97.9% compared to 2019 levels) was the most significantly affected by the pandemic, followed by subway (91.7%), buses (78.3%), and vehicle traffic volume for bridges and tunnels (65.5% by the end of May). In three regions of Sweden, which relied on recommendations instead of government mandates, public transport ridership was severely impacted (declining by 40% in Vastra and Gotland and 60% in Stockholm) (Jenelius and Cebecauer 2020). Public transit users changed their mobility patterns by switching from monthly period tickets to single tickets and travel funds (Jenelius and Cebecauer 2020; Orro et al. 2020) found that bus ridership in Coruña, Spain, was only 8–16% of 2017–2019 ridership. Lozzi et al. (2020) found that public transit dropped by 76% in April 2020 in 62 countries and 89 cities, compared to a baseline date of January 13, 2020.

**Table 3:** Overview of key studies on impact of COVID-19 on public transportation

Ref	Study area	Method	Data used	Mode	Key findings
Ahangari et al. (2020)	USA (10 medium-sized cities)	Comparative analysis, regression	Ridership & operation, sociodemographic data	Public transit	The ridership decreases from March, the start of the pandemic, while experienced the most decrease in ridership in April. The only factor affecting rail ridership reduction was the unemployment rate.
Almlof et al. (2021)	Sweden (Stockholm)	Binomial logit model	Smart card data	Public transit	Decreases in public transport use are linked to areas with a population of high socioeconomic status (e.g. income levels, owned houses and high employment levels).
Borsati et al. (2022)	Italy	Regression	COVID-19 mortality, transit usage	Public transit	Places with larger commuting flows exhibit higher excess mortality during the first wave of the pandemic, but no significant spatial association between excess mortality and transit usage.
Gkiotsalitis & Cats (2022)	USA (Washington DC)	Mixed-integer quadratic model	Metro operational data, O/D demand data	Public transit	It provides optimal redistribution of vehicles across lines for different social distancing scenarios.
Jenelius & Cebecauer (2020)	Sweden (3 cities)	Comparative analysis	Ticket validations, sales & passenger counts data	Public transit	The decrease in public transport ridership (40–60% across regions) was severe compared with other transport modes.
Jiang & Cai (2022)	China (Beijing, Shanghai)	Generalized linear models	Ridership, COVID-19, socioeconomic, weather	Metro	One additional cumulative local COVID-19 case within 14 days results in a reduction in metro ridership by 0.091% in Beijing & 0.112% in Shanghai.
Konecny et al. (2021)	Slovak	Analytical analysis	Population mobility, Demand & supply data	Public transit	The number of total passenger transport systems for suburban bus transport (SBT) decreased by 70% in April, 2020. There was a more significant decrease in the number of passengers in the first wave of the pandemic than during the second wave.
Liu et al. (2020c)	USA (113 public transit systems)	Logistic function model, regression & correlation	Transit mobile phone app, socioeconomic & demographic, COVID-19 case data	Public transit	Communities with higher proportions of essential workers, vulnerable populations (African American, Hispanic, Female, and people over 45 years old), and more coronavirus Google searches tend to maintain higher levels of minimal demand during the pandemic.
Orro et al. (2020)	Spain (Coruna)	Comparative analysis	Automatic vehicle location, bus stop boarding, smart card operation data	Car, bus, bicycle	The impact on transit ridership during the lockdown process was more significant than that on general traffic. These impacts are not uniform across the bus network.
Pozo et al. (2022)	Spain	Mathematical analysis	Ticket validation	Public transit	Ridership has dramatically decreased by 95% at the pandemic peak, recovering very slowly and reaching only half its pre-pandemic levels at the end of September, 2020.
Suman et al. (2020)	India (Delhi)	Optimization model	Bus demand & supply related data	Bus	The Business-as-Usual (BAU) scenario involving the current allocation approach will make it impossible to use public buses even if the bare minimum physical distancing has to be maintained.
Wilbur et al. (2020)	USA (Nashville, Chattanooga)	Comparative analysis	Ridership, census data	Public transit	Fixed-line bus ridership dropped by 66.9% and 65.1% from 2019 baselines before stabilizing at 48.4% and 42.8% declines respectively.
Yang & Chen (2022)	China	Regression	Daily operational frequency	Railway, aviation	HSR and aviation operations were both severely impacted by the outbreak of COVID-19. HSR generally has a strong substitution effect on the aviation system.

Air transportation was also severely impacted by COVID-19, because many countries implemented international travel bans. Commercial flight operations were dramatically reduced worldwide, with over two thirds fewer flights than in the same period in 2019 (Falchetta and Noussan 2020). Major airline carriers’ capacity dropped by 60–80% and airline industry job loss was estimated around 7% (Sobieralski 2020). Moreover, the study estimated that recovery from the adverse effects of the current uncertainty shock will take between four and six years. Iacus et al. (2020a) forecast air traffic volume and analyzed travel bans’ impact on the aviation sector using historical air traffic data, real time flight tracks, and online booking systems data.

Some studies investigated COVID-19’s heterogeneous impact on public transport users with different socioeconomic-demographic characteristics. A study shows that older people and female travelers are more likely to be conscious of COVID-19, while those who report using the train more often tend to be indifferent to infection (Shelat et al. 2022; Almlof et al. 2021) found that public transport use decreases were associated with income levels, house ownership, and high employment levels. Similarly, Liu et al. (2020c) found uneven impacts on transit systems and social groups in an analysis of 113 public transit systems in US communities. The study showed higher levels of transit demand during the pandemic in areas with higher proportions of essential workers, vulnerable populations (African American, Hispanic, female, and people over age 45), and more coronavirus Google searches. In a case study of Nashville and Chattanooga, TN, US, fixed-line bus ridership dropped by 66.9% and 65.1%, respectively, with a significant impact on low-income groups (Wilbur et al. 2020; Nikolaidou et al. 2023; Ahangari et al. 2020) investigated factors affecting public transport ridership, including the cleanliness of public transport, income inequality index, unemployment rate, poverty, education, and percentage of foreign-born residents.

The COVID-19 pandemic also led to changes in public transit services, where some public transport operators reallocated their services and provided minimum operations to meet essential travel demands, while considering government regulations and maintaining a safe transport mode (Limsawasd et al. 2022; Tiikkaja and Viri 2021; Meena 2020; Tirachini and Cats 2020; Ahangari et al. 2020). For example, Milan and Barcelona reduced vehicle occupancy to a maximum of 25% and 50%, respectively. Catalonia provided app users with bus occupancy levels in real time. The city of Hamburg adopted flexible bus routes to increase service on the busiest routes and reduce service frequency on lower demand routes (Lozzi et al. 2020). Gkiotsalitis and Cats (2022) and Suman et al. (2020) developed optimization models to redesign public transport services such as optimal service frequencies.

### Other impacts: bicycles, shared mobility, environment and traffic safety

As reviewed in previous sections, most research involved on case studies of the impact on personal and public transportation. Relatively few studies have examined the impacts of COVID-19 on other transport modes, such as bicycles and shared mobility. The usage behavior of these modes shows inconsistent results in each city, probably because the factors of decrease (e.g., decreased numbers of trips and increased working from home) and increase (e.g., effects of short-distance travel shifting from public transit) are mixed together. For example, during the pandemic, bike-sharing use decreased in London (Li et al. 2021b; Heydari et al. 2021), Lisbon (Teixeira et al. 2022), Bangkok (Sangveraphunsiri et al. 2022), and Slovakia (Kubal’ák et al. 2021), remained moderately stable in Korea (Choi et al. 2023), and increased in Singapore (Song et al. 2022) and Washington DC (Chen et al. 2022b). Ten cities in Germany also showed inconsistent results; the bicycle traffic volume decreased where the ratio of bicycle means was high and increased where the ratio of means was low, while pedestrian traffic decreased with higher local infectiousness and government measures (Mollers et al. 2022). In the case of London, shared bicycle usage immediately decreased due to the effect of the first lockdown but bicycle use increased during the lockdown period and showed a much larger increase after the first lockdown was lifted (Li et al. 2021b). Interestingly, morning peak travel and short-time travel by public bicycles in London maintained a low level of use during the lockdown and easing periods but were significantly higher at other times of the day and travel with middle and long duration. According to the study on the change in the travel behavior of bicycle sharing in Bangkok, shared bicycles were mostly used for business travel during the morning and afternoon peak hours on weekdays and leisure on weekends before COVID-19 (Sangveraphunsiri et al. 2022). However, the number of bicycle trips connecting subway stations in major university districts increased significantly after the pandemic.

There were also a few studies on changes in shared transportation and micro-mobility, and it was found that ridership was mostly decreased due to COVID-19 (Li et al. 2021c, d; Teixeira and Lopes 2020). For example, shared mobility ridership decreased by about 35% compared to normal in India (Meena 2020). In the case of Beijing, the overall share of shared mobility was kept constant between 36% and 38% both before and after COVID-19, but the proportion of ride-sharing decreased by 4.5% after COVID-19, while that of ride-hailing, car sharing, and bike sharing increased by 3.11%, 2.02%, and 0.89%, respectively.

Studies that examined COVID-19’s impact on environment and safety demonstrated that travel restrictions and reduced travel activities owing to COVID-19 resulted in improved air quality and safety (Llaguno-Munitxa and Bou-Zeid 2023; Nian et al. 2020; Muley et al. 2020; Cui et al. 2020; Sasidharan et al. 2020). Many studies have shown significant reductions in vehicle fuel consumption and emissions (Fischedick et al. 2021; Aloi et al. 2020). Vehicle emissions were estimated to decrease by 88.4% in 2020 and 48.6% in 2021 in Slovakia (Harantová et al. 2022) and by 14% in India (Advani et al. 2021). GHG emission was also estimated to decrease by 64% during the lockdown in Canada (Alama et al. 2022). COVID-19 and travel restriction policies have had a positive impact on traffic accidents, dropping by 67% in Santander, Spain (Aloi et al. 2020), 41% during the first month of COVID-19 in Greece, and 76% during the lockdown (March 16 – April 26, 2020) compared to 2018–2019 in Spain (Saladie et al. 2020).

### Changes in personal travel behavior based on surveys

The literatures reviewed in the previous chapter were mainly studies based on observed data. It is necessary to survey to analyze changes in personal travel behavior due to COVID-19 or the corresponding government’s measures. In this chapter, we review studies on this topic that were not revealed in aggregated data. Travel restrictions are effective tools for controlling infectious disease spread at the initial stages, while behavioral changes are important to limiting spread at a later stage (Muley et al. 2020). In addition to unprecedented total mobility reductions, the pandemic drastically impacted activity patterns and travel behavior through government implemented travel restrictions and individuals’ perceptions of safety and health (Table 4). These changes include transport mode choice (i.e., preferring more active and non-motorized modes), travel patterns (i.e., reducing non-essential trips and increasing work from home), and activity behavior (i.e., reducing outdoor activity and increasing online shopping) (Jou et al. 2022; Puelo, 2022; Nikiforiadis et al. 2022; Bhaduri et al. 2020; de Vos 2020; Moslem et al. 2020; Campisi et al. 2020; Borkowski et al. 2021; Tan and Ma 2021; Shamshiripour et al. 2020). Trip purpose, distance traveled, and trip frequency also changed (Abdullah et al. 2020). Significant predictors of mode choice included gender, car ownership, employment status, travel distance, primary purpose for travel, and pandemic-related underlying factors (Abdullah et al. 2020).

**Table 4:** Overview of key studies on impacts of COVID-19 on travel behavior changes

Ref	Study area	Method	Data used	Mode	Key findings
Abdullah et al. (2020)	Various countries	Descriptive & quantitative analyses	Survey	All types	Gender, car ownership, employment status, travel distance, purpose of traveling, & pandemic-related factors were found to be significant predictors of mode choice during the pandemic.
Abdullah et al. (2021)	Pakistan	Binary logistic model	Survey	Car, public transit	Gender, income, education, profession, trip frequency, car ownership, motorbike ownership, & safety precautions were found to be significant predictors of the public transit choice.
Astroza et al. (2020)	Chile	Joint models of probit & regression	Survey	All types	A decrease of 44% of trips in Santiago, with metro (55%), ride-hailing (51%), & bus (45%) present the highest reduction.
Atchison et al. (2021)	UK	Regression	Survey	All types	Ability to adopt & comply with certain non-pharmaceutical interventions is lower in the most economically disadvantaged in society.
Beck and Hensher (2020a)	Australia	Comparative analysis	Survey	Overall mobility	Aggregate travel has increased by 50% since initial restrictions, but is still less than two-thirds of that which occurred prior to COVID-19.
Beck & Hensher (2021b)	Australia	Descriptive analysis	Survey	Overall mobility	Reported trips have reduced significantly from an average of 23.9 trips per week down to 11.0, a reduction of over 50% in weekly household trips.
Bhaduri et al. (2020)	India	Multiple discrete choice model	Survey	All types	Found significant inertia to continue using the pre-COVID modes, & high propensity to shift to virtual (e.g. WFH) & private modes from shared ones (e.g. bus).
Borkowski et al. (2021)	Poland	General linear model	Survey	All types	Significant drops in travel times under epidemic conditions was observed, regardless of the age group & gender
Bounie et al. (2020)	France	Statistical analysis	Consumer transaction data	Overall mobility	The mandatory containment has significantly affected consumers’ mobility: visited fewer cities, & spent more in the home city.
Campisi et al. (2020)	Italy (Sicily)	Correlation & regression	Survey	All types	Women were less likely to walk than men. Participants were more likely to resume remote work even after the second phase.
Chan et al. (2020)	58 countries	Statistical analysis	Survey, Google reports, socio-demographic, COVID-19 cases	Overall mobility	Risk-taking attitudes are a critical factor in predicting reductions in human mobility & social confinement.
Costa et al. (2022)	Brazil	Multinomial & mixed logit models	Survey	All types	Comfort & frequency of the urban transit service were important factors to attract users during the pandemic.
Das et al. (2021)	India	Logistic regression	Survey	Car, public transit	Age, gender & monthly income tend to influence mode switch preferences. Travel time, overcrowding & hygiene are associated with mode shift preferences from public transport to car.
de Haas et al. (2020)	Netherlands	Comparative analysis	Survey	All types	Number of trips & distance travelled reduced by 55% and 68% respectively. About 80% of people reduced their activities outdoors, with a stronger decrease for older people.
Dingil & Esztergár-Kiss (2021)	International	Multinomial model	Survey	Overall mobility	Public transport users are 31.5, 10.6, and 6.9 times more likely to change their commuting transport mode than car users, motorcycle users, & walkers, respectively.
Downey et al. (2022)	Scotland	Bivariate probit model	Survey	Public transport	Over a third expect to use buses (36%) and trains (34%) less, whilst a quarter expect to drive their cars more.
Fatmi (2020)	Canada (Kelowna)	Comparative analysis	Daily activities survey	Overall mobility	Individuals’ participation in out-of-home activities were reduced by more than 50%. The majority of long-distance travel was made regionally using private car.
Harrington & Hadjiconstantinuou (2022)	UK	Statistical analysis	Survey	All types	Of the car commuters, 81.9% may continue travelling by car once restrictions are lifted, while of the public transport commuters, 49.0% might switch modes.
Hotle et al. (2020)	USA	Generalized ordered logit regression	Survey	All types	A recent personal experience with influenza-like symptoms & being female significantly increased risk perception at mandatory & medical trip locations.
Javadinasr et al. (2022)	USA	Ordered probit model	Survey	All types	48% of the respondents anticipate having the option to WFH after the pandemic, which indicates an approximately 30% increase compared to the pre-pandemic period. In the post-pandemic period, auto and transit commuters are expected to be 9% and 31% less than pre-pandemic, respectively
Jiao & Azimian (2021)	USA	Binary logit model	Survey	All types	Age, gender, educational status, marital status, work loss, difficulty with expenses, household size, work type, income, health status, & anxiousness were associated with changes in travel behavior.
Jou et al. (2022)	Taiwan	Logistic regression, ordered logit models	Survey	All types	The total travels by private vehicles are significantly reduced, but no significant decrease in the use of transit, possibly because transit users have no choice.
Kamplimath et al. (2021)	India	Statistical analysis	Survey	All types	Comfort & hygiene are now the most important factor that affects the mode choice of travel followed by the cost and travel time.
Marra et al. (2022)	Switzerland	Comparative analysis, Mixed logit model	Travel survey (GPS tracking)	Public transit	The travel distance for every mode of public transit in 2020 is around 50% less than 2019.
Mashrur et al. (2022)	Canada	Logit choice models	SP survey	Public transit	Transit frequency dropped by 21–71% for various socioeconomic groups. Vaccine availability & mandatory face-covering onboard positively affect choices of riding transit.
Meena (2020)	India	Descriptive analysis	Survey	All types	After the end of lockdown, people will reduce their non-mandatory trips & higher income group will try to avoid travelling in public transport, taxi & other mass transport.
Meister et al. (2022)	Switzerland	Mixed discrete-continuous model	GPS tracking travel diary data	All types	Public transit saw the largest decrease in traveled distance & trip frequencies, with an almost 100% reduction during lockdown.
Mogaji (2020)	Nigeria (Lagos)	Statistical analysis	Survey	Overall mobility	The study recognizes the effect on transportation in emerging economies, where lockdowns and restrictions on movement may be ineffective.
Moslem et al. (2020)	Italy (Palermo, Catania)	Best-worst method	Survey	All types	Bus remained the third choice of Italians, but the multimodality increased, which may influence the mobility choices even if the epidemic ends.
Parady et al. (2020)	Japan	Regression	Survey	All types	Risk perception was associated with higher probabilities of going-out self-restriction for eating-out and leisure.
Pawar et al. (2020)	India	Decision tree analysis	Survey	All types	About 41% of commuters stopped traveling, 51.3% were using the same mode of transport & 5.3% of commuters shifted from public to private mode. Safety perceptions did not play a significant role in mode choice behavior.
Przybylowski et al. (2021)	Poland (Gdansk)	Descriptive analysis	Survey	All types	About 90% of respondents resigned or limited their usage of public transport. Almost 75% of respondents plan to return to using public transport when the epidemic situation has stabilized.
Schaefer et al. (2021)	German (Hanover)	Regression	Survey	All types	Local light rail & bus are substituted by bike, car & WFH, while train use is not significantly replaced by car & seems to be positively related to bike use.
Shakibaei et al. (2020)	Turkey (Istanbul)	Descriptive analysis	Panel data	All types	5.6% of the commuters who were using public transit during phase 1 of the study started to use private car during phase 2. Shift to the private car was even more remarkable in the transition to phase 3.
Shelat et al. (2022)	Netherlands	Latent class choice model	SP survey	Railway	Older and female travellers are more likely to be COVD conscious while those reporting to use the trains more frequently tend to be infection indifferent.
Simovi´c et al. (2021)	7 South-East European countries	Regression	Survey	Public transit	The acceptability of vehicle occupancy differs with respect to age, education, & health conditions of the respondents.
Sogbe (2021)	Ghana	Statistical analysis	Survey	Public transit	Commuters considered physical distancing, occupants wearing face masks, cleanliness of vehicle & safety as essential factors for transit mode choice.
Tan & Ma (2021)	China	Logistic regression	Survey	Rail	Occupation, commuting modes before COVID-19, walking time to the nearest subway station, the possibility of being infected in private car in public transport have significant influence on the commuters’ choice of rail transit.
Yang et al. (2021)	China	Qualitative analysis	Survey	All types	Students, lower income cohorts, groups living in small communities, & those working in tourism, catering, informal businesses & transport-related sectors were more vulnerable than others.
Zubair et al. (2022)	Thailand	Regression	Survey	All types	People’s priorities shifted from travel time saving, safety & security, comfort, & cleanliness to infection concerns, social distance, cleanliness, & passengers’ face masks for mode selection.

Many studies conducted preference surveys and demonstrated significant travel behavior changes with different perspectives and impacts on sociodemographic groups (Ferreira et al. 2022; Javadinasr et al. 2022; Szczepanek and Kruszyna 2022; Downey et al. 2022; Zhou et al. 2022; Currie et al. 2021; Echaniz et al. 2021; Abdullah et al. 2020; Morita et al. 2020; Tan and Ma 2021; Shakibaei et al. 2020; Ghader et al. 2020; Przybylowski et al. 2021; Pan et al. 2020). All survey results indicated that the pandemic impacted mode choice behavior, with people avoiding crowded places to maintain social distance, which resulted in significantly reduced public transport and shared mobility demand owing to health concerns, and increased dependence on private vehicles (Oestreich et al. 2023; Nian et al. 2020). In Santiago (Astroza et al. 2020), overall trips were reduced by 44% (with the highest reduction in metro (55%), ride-hailing (51%), and bus (45%)). Transport modes relatively less affected by COVID-19 were motorcycle (28%), auto (34%), and walking (39%). While 77% of workers from low-income households had to go out to work, 80% of workers from high-income households worked from home. In the UK, 81.9% of private commuters responded that they would continue to use their car even when restrictions are lifted, while only 3.6% and 6.5% said they could switch to walking and biking, respectively (Harrington and Hadjiconstantinou 2022). On the other hand, public transportation users from diverse locations in the world were 31.5, 10.6, and 6.9 times more likely to change their commuting mode than car users, motorcycle users, and pedestrians, respectively (Dingil and Esztergár-Kiss 2021; Bhaduri et al. 2020) analyzed the effect of traveler’s sociodemographic characteristics on travel mode choice. About 95% of respondents said that both their daily commute and discretionary travel behavior were affected by the pandemic. Meena (2020) analyzed the impact of COVID-19 on travel patterns during normal, pre-lockdown, and post lockdown periods and found that private car use increased during pre-lockdown (21%) and was expected to increase more significantly during the post lockdown period (31%), compared to the normal situation (17%). Although there were differences in degree, mode choice changes were similarly observed in other surveys, including Palermo and Catania in Italy (Moslem et al. 2020), Istanbul in Turkey (Shakibaei et al. 2020), Gdansk in Poland (Przybylowski et al. 2021), and China (Tan and Ma 2021). The COVID-19 pandemic has also changed the factors influencing mode choice (Das et al. 2021). Prior to COVID-19, factors such as travel time saving, safety, security, and comfort (Zubair et al. 2022) and factors including distance and duration of travel (Mussone and Changizi 2023) were important influencing factors in the choice of modes, but during the pandemic, concerns about infection, social distancing, wearing a mask, and worry about using public transport became important influencing factors (Zubair et al. 2022; Mussone and Changizi 2023). Studies also show that comfort and frequency of transit (Costa et al. 2022), the availability of vaccines, and the obligation to wear a mask onboard (Mashrur et al. 2022) affect the choice of public transit. Mancinelli et al. (2022) investigated a change in travel patterns departing from airports and ports. They found that before COVID-19, about 73% of respondents used public transportation as an accessibility mode, but the proportion was less than 50% during the pandemic, and the intention to use public transportation after COVID-19 surveyed to be about 56% (Mancinelli et al. 2022).

While many studies focused on mode choice behavior changes related to the pandemic, other studies investigated travel characteristics, such as travel distance. In Switzerland, compared to 2019, the travel distance of all means of transportation decreased by 50% at the beginning of the outbreak, and when the first restriction was implemented, the travel distance of public transportation decreased by more than 90% (Hintermann et al. 2023; Marra et al. 2022; Meister et al. 2022). Using a survey distributed in various countries, Abdullah et al. (2020) found that the percentage of respondents who traveled for a short trip (a distance less than 10 km) dropped from 71% before the pandemic to 45% during the pandemic. The average work trip distance was 3.6 km and 2.6 km before and during the pandemic, respectively. In fact, these numbers are much smaller than expected, probably due to the analysis of diverse countries, including underdeveloped countries. Travel distance differences before and during the pandemic were also reported by other studies (Borkowski et al. 2021; Bounie et al. 2020; De Haas et al. 2020).

Travel behavior is a complex issue, influenced by various factors such as sociodemographic and personal characteristics (Simovi´c et al. 2021; Abdullah et al. 2021; Jiao and Azimian 2021; Borkowski et al. 2021). When investigating the mode choice behavior before and during the pandemic, Abdullah et al. (2022) found that during the pandemic, monthly household income and epidemic-related factors were important predictors for short-distance (i.e., < 5 km) mode choice, whereas gender, car ownership, and monthly household income were significant predictors for longer distances (i.e., > 5 km). In a survey administered in Sicily, Italy, women were 1.5 times more likely to reduce walking frequency than men (Campisi et al. 2020; De Haas et al. 2020) found that about 80% of respondents in the Netherlands panel data reduced their outdoor activities. In particular, older people tended to reduce activities more than before the pandemic. Travel behavior changes in terms of out-of-home travel activities, activity purposes, and travel differences by income level were also observed in Canada (Fatmi 2020). Respondents in Lagos, Nigeria showed a positive correlation between transportation influenced by COVID-19 and its impact on economic (correlation coefficient of 0.442), social (0.313) and religious (0.274) activities (Mogaji 2020).

Working from home (WFH) increased, emerging as one of the government policies during the pandemic (Hensher et al. 2022, 2023; Ecke et al. 2022; Mouratidis and Peters 2022; Balbontin et al. 2022; Beck and Hensher 2020a, b). About 71% of Chicago US respondents reported that they had not experienced working from home before the pandemic, while about 63% reported that they did experience working from home during the pandemic (Shamshiripour et al. 2020). The value of travel time has changed due to the WFH policy, increasing by 12.55% compared to before the pandemic in Australia (Hensher et al. 2021). Using GPS tracking data in Switzerland, Huang et al. (2023) found more significant reductions of trip distance, travel time, travel frequency, morning peak hours trips, and trips to the CBD among the WFH group. Promoting WFH also decreased traffic congestion, especially during morning peak hours, in Hong Kong (Loo and Huang 2022). The main factor influencing WFH during the lockdown period in the Netherlands was job characteristics; office workers and teaching staff were more likely to spend more time working from home (Kalter et al. 2021). In a study analyzing WFH patterns using data from eight countries, the results show that the role of socioeconomic characteristics differs from country to country (Balbontin et al. 2021). In South America, for example, older adults and women are more likely to have WFH compared to other countries analyzed, and income has a positive effect on the number of WFH days in Australia and Chile. However, an issue of inequity was revealed as low-income and low-educated people were mainly unable to WFH and did not have flexible working hours (Ecke et al. 2022).

Fear of contagion and perceived risk also significantly impacted travel patterns (Airak et al. 2023; Navarrete-Hernandez et al. 2023; Zavareh et al. 2022; Aghabayk et al. 2021; Przybylowski et al. 2021; Abdullah et al. 2020). Awareness of overcrowding during the COVID-19 pandemic is about 1.04 to 1.23 times higher than before the pandemic (Cho and Park 2021). Women tend to be more sensitive than men to fear of infection and the use of face masks on public transport (Basnak et al. 2022; Schaefer et al. 2021). On the other hand, younger and low-income people are relatively less sensitive to overcrowding (Basnak et al. 2022). When exploring risk perception effects on human mobility for 58 countries using Global Preferences Survey data, Chan et al. (2020) found that regions with risk-averse attitudes were more likely to adjust their mobility behavior in response to the WHO declaration of a pandemic even before official government lockdowns. Przybylowski et al. (2021) found that willingness to use public transport depended mostly on perceived comfort and safety during the pandemic. Parady et al. (2020) examined pandemic related factors affecting behavioral changes in non-work-related activities in Japan, which focused on the effects of risk perception and social influence. Yuksel et al. (2020) conducted a case study in Canada that examined behavioral parameters of change in mobility and sentiment that reflected people’s beliefs about how contagious the disease is on the level of compliance with public orders. Mode choice behavior changes might be maintained for a long time owing to concerns about infection risk (Nian et al. 2020). Although Hotle et al.‘s (2020) survey was not conducted during the COVID-19 pandemic, the authors found that a recent personal experience with influenza symptoms resulted in higher risk perception at mandatory and medical trip locations in women, while men were not likely to change their travel patterns in response to potential virus spread or increasing exposure. Interestingly, high perceived workplace risk did not significantly reduce individuals’ travel to their workplaces. In addition, when Pawar et al. (2020) investigated the impact of COVID-19 on mode choice during the transition to a lockdown period in India, they found that commuters’ safety perceptions did not have a significant effect on transportation mode choice.

## Effects of measures on mobility reduction and COVID-19 spread

The COVID-19 pandemic presented an unprecedented challenge to governments, forcing them to implement various non-pharmaceutical countermeasures to reduce the possibility of contact and minimize disease transmission. Such interventions included complete city lockdowns, travel restrictions, stay-at-home policies, some location closures, and social distancing policies Rosik et al. 2022; Zhang et al. 2021a; Gkiotsalitis and Cats, 2021; Gao et al. 2020c; Yabe et al. 2020; Wielechowski et al. 2020; Schwartz 2020a). Numerous countries introduced different types and degrees of restrictive policies (e.g., from complete lockdown in China, lockdown in Italy, Spain, and France, to mild and less restrictive policies in Sweden, Netherlands), which influence people’s lifestyles, social interactions, travel behaviors, and activity behaviors (Borkowski et al. 2021; Abdullah et al. 2020, de Haas et al. 2020; Klein et al. 2020a>; de Vos 2020).

The impact of such interventions on transportation systems, travel behavior, and COVID-19 spread has drawn much research attention (Table 5). According to Jaekel and Muley (2022), reduced traffic volumes were more associated with restrictive measures than COVID-19 incidences in both Germany and Qatar. However, the relationships between the measures and travel behavior changes in response to COVID-19 are complex. Glaeser et al. (2022) emphasized that evaluating the effectiveness of restrictions on mobility is challenging for several reasons: the restriction policies are adopted to limit the spread of outbreaks, while individuals make decisions on travel based on their personal attitudes regarding risk of contagion. It is also important for policy makers to understand the efficacy of restriction policies in any given time and region to prepare for future disease outbreaks (Yuksel et al. 2020). Accordingly, this chapter reviews studies explicitly, analyzing the effects of various implemented policies, and discusses them under three topics.

**Table 5:** Overview of key studies on effects of measures on mobility

Ref	Study area	Type of measure^※^	Method	Data used	Mode	Key findings
Aloi et al. (2020)	Spain (Santander)	A	Comparative analysis	Traffic count, public transport data	Car, public transit	Public transport users dropped by up to 93%, NO2 emissions were reduced by up to 60%, & traffic accidents were reduced by up to 67% in relative terms.
Anzai et al. (2020)	China, Japan	C	Statistical model	COVID-19 cases, travel volume	Overall mobility	As the delay is small, the decision to control travel volume through restrictions on freedom of movement should be balanced between the resulting estimated epidemiological impact and predicted economic fallout.
Arellana et al. (2020)	Colombia (7 cities)	A, F	Comparative analysis	Traffic volume & operation data, Google reports	Air, freight, urban transport	National policies & local decisions have decreased motorized trips, diminishing congestion levels, reducing transit ridership, & creating a reduction in transport externalities.
Askitas et al. (2020)	135 countries	C, D	Multiple events model	COVID-19 prevalence data, Google reports	Overall mobility	Cancelling public events & enforcing restrictions on gatherings have the largest effect on curbing the pandemic. Workplace & school closures as well as stay-at-home requirements also reduce activities away from home, but not as large as for public events and gatherings.
Awad-Núñez et al. (2021)	Spain	C	Choice modeling	Survey	Public transport, shared mobility	Some measures, such as the increase of supply & vehicle disinfection, result in a greater willingness to use public transport in post-COVID-19 times.
Buhat et al. (2020)	Philippines (Manila)	F, G	Agent-based model simulation	N/A	Train, bus	Social distancing reduces the risk of being infected; minimizing movement or interaction with other passengers reduces the risk of transmission by 50%; passenger capacity should be less than 10–50% of the maximum seating capacity to reduce the number of infections.
Chen et al. (2022a)	Netherlands	Various (Four-level)	Error component latent class choice model	Survey	Public transport	The older & highly educated people are more susceptible to enforcement measures, whereas young & single citizens are more accessible to noncompulsory measures.
Chen & Pan (2020)	China	B	Comparative analysis	National & global epidemic data	Overall mobility	Social distancing is important for controlling the spread of the epidemic.
Chinazzi et al. (2020)	China	C	Epidemic & mobility model	COVID-19 case, airline flow, ground mobility flow	Overall mobility	The travel quarantine of Wuhan delayed the overall epidemic progression by only 3 to 5 days in mainland China but had a more marked effect on the international scale, where case importations were reduced by nearly 80% until mid-February.
Dahlberg et al. (2020)	Sweden (Stockholm)	F	Difference-in-difference	Mobile phone data	Overall mobility	The daytime population in residential areas increased significantly (64%). The distance individuals move from their homes during a day was substantially reduced (38%).
Dasgupta et al. (2020)	USA	F	Regression	Mobile device location, health & socioeconomic related data	Overall mobility	Counties without stay-at-home orders showed a mobility decline of -52.3%, slightly less than the decline in mandated areas (-60.8%).
Espinoza et al. (2020)	N/A	C	Disease transmission model & simulation	COVID-19 infection risk & community-specific characteristics	Overall mobility	Mobility restrictions may not be an effective policy for controlling the spread of an infectious disease if it is assessed by the overall final epidemic size.
Fang et al. (2020a)	China	C	SEIR model	COVID-19 case data	Overall mobility	More rigorous government control policies were associated with a slower increase in the infected population. Isolation & protective procedures would be less effective as more cases accrue.
Fang et al. (2020b)	China	A*	Difference-in-differences	Population migration, COVID-19 infection data	Overall mobility	The lockdown of Wuhan reduced inflows to Wuhan by 76.98%, outflows from Wuhan by 56.31%, and within-Wuhan movements by 55.91%.
Galeazzi et al. (2021)	France, Italy, UK	A	Analytical calculation	Facebook data, mobility network	Overall mobility	The reduction of the overall efficiency in the network of movements is accompanied by geographical fragmentation with a massive reduction of long-range connections.
Gao et al. (2020b)	USA	F	Statistical analysis	Smartphone location data	Overall mobility	The platform provides daily mobilities in terms of median travel distance, percent change in mobility, & home dwell time.
Gao et al. (2020c)	USA	F	Regression	Mobile phone location, COVID-19 case	Overall mobility	The correlation between the COVID19 growth rate and travel distance decay rate & dwell time at home change rate was − 0.586 & 0.526, respectively.
Ghader et al. (2020)	USA	F	Comparative analysis	Mobile location, COVID-19 case, census population	Overall mobility	Statistics related to social distancing, namely trip rate, miles traveled per person, and percentage of population staying at home have all showed an unexpected trend, which we named “social distancing inertia.”
Gramsch et al. (2022)	Chile	A	Regression	Smartcard data	Public transport	A decrease of 72.3% when schools suspended in-person classes, while the dynamic lockdowns reduced public transport demand by 12.1%. The effect of lockdowns decreased after the fifth week of their application.
Hadjidemetriou et al. (2020)	UK	A, C	Logistic & regression models	Apple reports, COVID-19 related death	Car, transit, walking	Human mobility was observed to gradually decrease as the government was announcing more measures and it stabilized at a scale of around 80% after a lockdown was imposed.
Heiler et al. (2020)	Austria	A	Comparative analysis	Mobile phone, COVID-19 infection data	Overall mobility	A reduction of commuters at Viennese metro stations of over 80% & the number of devices with a radius of gyration of less than 500 m almost doubled.
Jaekel & Muley (2022)	Germany, Qatar	C	Comparative analysis	Google reports, traffic volume & crashes data	Overall mobility	The reduction in traffic volumes, major & minor crashes was coupled with restrictive measures rather than COVID-19 incidences for both countries.
Klein et al. (2020a)	USA	D	Comparative analysis	Mobile device location	Overall mobility	By March 23, 2020 the policies have generally reduced by half the overall mobility in several major U.S. cities.
Klein et al. (2020b)	USA	D	Data analysis	Mobile device location, commute data	Overall mobility	The average person had reduced their daily mobility by between 45–55% as of late April, 2020 and had reduced their daily contacts between 65–75%.
Kraemer et al. (2020)	China	C	Generalized linear model	Real-time mobility, COVID-19 case & demographics	Overall mobility	Early on, the spatial distribution of COVID-19 cases in China was explained well by human mobility data. But, after the implementation of control measures, this correlation dropped and growth rates became negative in most locations.
Linka et al. (2020)	Europe	C	SEIR model	Passenger air travel statistics, COVID-19 case data	Air	Mobility networks of air travel can predict the emerging global diffusion pattern of a pandemic at the early stages of the outbreak.
Martin-Calvo et al. (2020)	USA (Boston)	F	SEIR model	Mobility data, census, COVID-19 data	Overall mobility	School closures & passive social distance strategies are not enough to contain the epidemic. A full confinement is not feasible & will not solve the problem, without active measures in place after the confinement, since there would be a new outbreak.
Morita et al. (2020)	Japan (4 cities)	F	Correlation analysis	Google & Apple reports	Car, transit, walking	The behavioral inhibition manifests differently de-pending upon urban structure and climatic factors.
Muller et al. (2020)	Germany (Berlin)	A	Dynamics model & simulation	Activity chains & trajectory	Overall mobility	Complete lockdown works. Complete removal of infections at primary schools, workplaces & during leisure activities will not be enough to sufficiently slow down the infection dynamics. Infections in public transport play an important role.
Oum & Wang (2020)	N/A	A, C	Economic model	N/A	Overall mobility	Individuals do not internalize the external cost of infection risks they impose on others when making their own decisions, implying that the socially optimal length of lockdown is always longer than the privately optimal length of the lockdown period.
Pan et al. (2020)	USA	D, F	Comparative & correlation	Mobile device location	Overall mobility	Both government orders & local outbreak severity significantly contribute to the strength of social distancing.
Park (2020)	Korea (Seoul)	F	Statistical analysis	Subway ridership, COVID-19 cases	Subway	Compared to the third week of January 2020, the mean daily number of passengers in all stations decreased by 40.6% by the first week of March.
Pepe et al. (2020)	Italy	A	Analytical analysis	Mobile phone location, Google reports	Overall mobility	Daily time-series of three different aggregated mobility metrics can help to monitor the impact of the lockdown on the epidemic trajectory & inform future public health decision making.
Pullano et al. (2020)	France	A	Correlation analysis	Mobile trajectory, hospitalization, socioeconomic data	Overall mobility	Lockdown caused a 65% reduction in countrywide number of displacements. Individual response to policy announcements may generate unexpected anomalous behaviors increasing the risk of geographical diffusion.
Santamaria et al. (2020)	Europe (15 countries)	A	Analytical calculation	Mobile positioning	Overall mobility	A large proportion of the change in mobility patterns can be explained by confinement measures.
Schlosser et al. (2020)	Germany	A	Analytical SIR model	Mobile phone data	Overall mobility	Long-distance travel was reduced disproportionately strongly. The structural changes have a considerable effect on epidemic spreading processes.
Vannoni et al. (2020)	41 cities worldwide	C	Multivariate models	Mobility index, Oxford COVID-19 response dataset	Overall mobility	After adjusting for time-trends, the study observed that implementing non-pharmaceutical countermeasures was associated with a decline of mobility of 10.0% for school closures, 15.0% for workplace closures, 7.09% for cancelling public events, 18.0% for closing public transport.
Wei et al. (2021)	China	A, C	Epidemic & mobility model	Population mobility, COVID-19 case, city network	Overall mobility	The containment effect of the lockdown of cities in Hubei was greater than that of decreasing intercity population mobility, & the effect of city lockdowns was more sensitive to timing relative to decreasing population mobility.
Wellenius et al. (2021)	USA	F	Regression	Google reports	Overall mobility	State-of-emergency declarations resulted in a 10% reduction in time spent away from places of residence. Implementation of one or more social distancing policies resulted in an additional 25% reduction in mobility.
Wielechowski et al. (2020)	Poland	A, C	Statistical analysis	Google reports, COVID-19 Response Tracker	Public transit	There is negative but insignificant relationship between human mobility changes in public transport & the number of new confirmed COVID-19 cases.
Xu et al. (2020)	USA	F	Comparative & correlation	Twitter	N/A	A large reduction in travel after the implementation of social distancing policies, with larger reductions in states that were early adopters and smaller changes in states without policies.
Yabe et al. (2020)	Japan (Tokyo)	C	Comparative analysis	Mobile phone, socioeconomic data, COVID-19 case	Overall mobility	Human mobility behavior decreased by around 50%, resulting in a 70% reduction of social contacts, showing the strong relationships with non-compulsory measures.
Yilmazkuday (2020)	USA	E	Difference-in-difference	Travel data (smartphone), COVID-19 cases & deaths	Overall mobility	Staying in the same county has the potential of reducing total weekly COVID-19 cases and deaths as much as by 139,503 and by 23,445, respectively.
Zhang et al. (2020a)	China	A*	SIR model	Population flow, daily infection data	Overall mobility	The study supports the existence of non-lock-down-typed measures that can reach the same containment consequence as the lock-down.
Zhang et al. (2021a)	USA	F	Data integration & analysis	Mobile location, COVID-19 case, census population	Overall mobility	The interactive analytical tool identifies trips & produces a set of variables including social distancing index, percentage of people staying at home, visits to work and non-work locations, out-of-town trips, & trip distance.
Zuo et al. (2020)	USA (NY, Seattle)	F	Data mining	COVID-19 case, transportation related data	Overall mobility	The mobility board presents multi-data views in terms of vehicular traffic volume, corridor travel time, transit ridership, freight traffic, as well as risk indicators in terms of reported crashes, pedestrian and cyclist fatalities & speeding tickets.

※ A* (lockdown in China), A (lockdown in other countries), B (strict travel restriction), C (travel restriction), D (stay-at-home), E (saying in the same county), F (social distancing), G (limiting capacity and density of transit)

### Travel restriction policies’ impacts on reducing human mobility

During the pandemic, most cities around the world are experiencing a decrease in traffic volume, resulting from a combination of restrictive policies rather than the impact of the outbreak itself (Jaekel and Muley 2022), because many epidemic prevention and control policies involve travel and activity restrictions. Global statistical data indicated that the restriction policy has substantially reduced transport demand. In particular, China’s city lockdown policy is unprecedentedly strong in the world, showing that it has the effect of controlling traffic and preventing the spread of COVID-19. For example, the Wuhan lockdown reduced inflows by about 77%, outflows by about 56%, and within-Wuhan movements by about 56% (Fang et al. 2020b). In addition, without the Wuhan lockdown, it was estimated that the number of positive COVID-19 cases would be 105% higher (Fang et al. 2020b). Although not as strong as China, several other countries have implemented city lockdowns and have shown effectiveness in controlling traffic (Jaekel and Muley 2022; Mars et al. 2022; Hadjidemetriou et al. 2020): that is, lockdown restrictions reduced (1) human mobility by 65% in France (Pullano et al. 2020), (2) mobility rate by 74.2% (21.7 trips/week before the pandemic vs. 5.6 trips/week during the lockdown) in Spain (Mars et al. 2022), and (3) long-distance travel in Germany (Schlosser et al. 2020).

However, several case studies show that a lockdown is not the only effective means of reducing traffic volume. According to the study analyzing public transport demand in Chile using smart cards (Gramsch et al. 2022), when the first measures (e.g., schools suspended in-person classes) were implemented at the beginning of the pandemic, the demand for public transport decreased by 72.3% compared to the year 2019, while it decreased by 12.1% with the dynamic lockdown implemented by each city. In particular, the effect of the lockdown decreased five weeks after its implementation, suggesting that the lockdown policy effectively controls the traffic volume in a short period of time. In this sense, the results of Dahlberg et al. (2020)’s study analyzing Sweden’s less restrictive policy are interesting. When using mobile phone data to investigate COVID-19 causal effects, they found that even less restrictive and mild public recommendations convince people to comply with social distancing and avoid unnecessary travel (i.e., residential area daytime population increased by 64%; industrial and commercial area daytime population decreased by 33%; travel distance decreased by 38%; share of short trips less than one kilometer from home increased by 36%; and mobility change effects did not differ across socioeconomic and demographic characteristics). In addition, when comparing lockdown measure effects on mobility patterns in France, Italy, and the UK, Galeazzi et al. (2021) found that their mobility patterns differed in response to the travel restrictions owing to differences in existing infrastructure characteristics and initial mobility structure.

Besides strong restriction measures, such as city lockdowns and travel restrictions, several studies investigated the effects of less restrictive or non-compulsory policies. When examining the mobility impact of different non-pharmaceutical countermeasures for 41 cities worldwide, Vannoni et al. (2020) found that the decrease in mobility is 18% due to closing public transport, 15% due to workplace closures, 13.3% due to restricting internal movements, 10% due to school closures, and 7.09% due to canceling public events. In Tokyo, non-compulsory policies, such as remote working with private companies and school closures, reduced human mobility and social contact by about 50% and 70%, respectively (Yabe et al. 2020). Similarly, government interventions reduced overall mobility by about 50% in several major US cities (Klein et al. 2020b) and reduced all station ridership by about 40.6% in Seoul, Korea (Park 2020). National social distancing measures were effective for intra-city vehicle movement, particularly at night, but not for inter-city movement in Korea (Sung 2022). Stay-at-home orders in the U.S. and Japan also showed a positive effect in reducing mobility (Liu and Yamamoto 2022; Gao et al. 2020c). Analysis using mobile phone data showed that counties without stay-at-home orders in the U.S. reduced mobility by 52.3%, while counties with stay-at-home orders experienced a slightly larger mobility drop at 60.8% (Dasgupta et al. 2020).

The social distancing policy of maintaining at least six feet between people emerged as a widely accepted non-pharmaceutical intervention to mitigate the pandemic (Chen et al. 2022a; Liu et al. 2022; Vichiensan et al. 2021; Zhang et al. 2020b; Morita et al. 2020). Although social distancing might negatively affect subjective well-being and limit physical activity, many studies supported the positive effects on travel behavior to prevent social contact and COVID-19 spread (De Vos 2020; Fang et al. 2020b). The public geo-located US Twitter data showed a significant 61.83% overall travel reduction after social distancing policies were implemented (Xu et al. 2020). In particular, larger reductions were found in states that were early adopters of social distancing practices, whereas smaller changes were found in states without such policies. Analysis using Google Community Mobility reports in the US showed that state-of-emergency declarations had only a modest effect on mobility (about a 10% decrease), but implementing one or more social distancing policies resulted in an additional 25% mobility decrease (Wellenius et al. 2021).

The implications of the studies discussed above are: (1) the effects of coercive policies are effective in the short-term (Liu et al. 2022); (2) policy announcement and implementation timing are important because unexpected anomalous behaviors can occur (Pullano et al. 2020; Liu et al. 2020b); and (3) it is more effective to introduce a combination of different types of control policies (Wang et al. 2022; Chinazzi et al. 2020; Anzai et al. 2020; Wellenius et al. 2021).

### The relationship between travel restriction policy and COVID-19 transmission

Governments implemented control and prevention policies to decrease traffic volume and person-to-person contact, which ultimately lead to reduced disease spread. Various studies examined the effectiveness of measures developed to limit COVID-19 spread and found that travel restrictions and social distancing directly affected travel behaviors, thus, effectively slowing COVID-19 spread, but at different levels (Chen et al. 2022a; Wang et al. 2022; Manzira et al. 2022; Espinoza et al. 2020; Zhang et al. 2020b; Chen and Pan 2020). The number of daily COVID-19 cases in Italy was directly associated with trips taken three weeks before (Carteni et al. 2020). In addition, the population outflow distribution significantly influenced the spatiotemporal distribution of confirmed COVID-19 cases in Wuhan, China, and the authors argued that the effect of quarantines on mobility to limit COVID-19 transmission was obvious (Jia et al. 2020).

There have also been studies showing that local travel restrictions were effective for controlling COVID-19 infection. In China, where the spread of COVID-19 was most severe, the suspension of high-speed rail and air connectivity with Wuhan reduced the number of daily new confirmed cases by 18.6% and 13.3%, respectively (Zhu and Guo 2021). According to the scenario simulation results using data from China, if lockdown and decreased population mobility policies were not implemented, the total number of infectious cases would have reached 138,824 in February 2020, corresponding to 4.46 times the actual case number (Wei et al. 2021). Travel restrictions implemented by local cities outside Hubei also decreased confirmed cases by 22.4% in the first two weeks after the Wuhan lockdown (Liu et al. 2020b). Without intra-city travel restrictions, the confirmed cases were estimated to increase by 33.1%. Based on these results, the authors asserted that if travel restrictions were implemented in advance in the entire Hubei province, the number of confirmed cases might have decreased by another 10.5%, emphasizing the importance of a timely and coordinated response to mitigate the pandemic. Another study found that travel restrictions may have reduced expected cumulative incidence by 39% in Wuhan by February 29, 2020 (Shi and Fang 2020). Staying in the same county also effectively limited COVID-19 cases and deaths. US data showed that staying in the same county reduced total weekly COVID-19 cases by 139,503, and deaths by 23,445 (Yilmazkuday 2020).

Other studies suggest that simply implementing one restriction measure does not have a significant effect on decreasing new infections. According to Chinazzi et al.’s (2020) transmission model, 90% travel restrictions to and from mainland China only modestly affected pandemic spread, delaying it for two weeks at best, unless it was combined with a strong reduction (i.e., 50% or higher) in community transmission. China and worldwide data analyzed with the susceptible-exposed-infectious-recovered (SEIR) model showed that more rigorous government control policies were associated with a slower infection rate, and isolation and quarantine procedures were less effective for controlling the pandemic (Fang et al. 2020a). When estimating the impact of travel restrictions, including lockdown in Wuhan, China, on COVID-19 incidence, Anzai et al. (2020) found that the estimated delay was smaller than the authors expected depending on the scenario. Therefore, they argued travel restriction decisions, such as a complete lockdown, should be carefully applied by comparing the resulting estimated epidemiological impact and predicted economic outcomes. Pan et al.’s (2020) analysis of mobile phone location data showed a similar trend. The authors proposed a social distancing index, which indicated that both government orders and local outbreak severity were significantly associated with the strength of social distancing.

### Controversial issues related travel restriction policy effects

Since the COVID-19 outbreak, many studies have examined and explained the effectiveness of government control and prevention measures. However, the effectiveness of diverse measures has been a subject of debate (Anzai et al. 2020) because studies report inconsistent results or findings showing less effectiveness than expected, and identify controversial issues, such as control strategy side effects. Moreover, it is difficult to quantify and distinguish measure effects from other potential contributing factors (Liu et al. 2022; Fang et al. 2020b). It may be unreasonable to draw one conclusion based on a single standard in this study because the timing and method of government policy implementation, citizen compliance, and analysis data and methodologies are different in each city. Nevertheless, it is of great significance to review the research results published so far and to learn some lessons. Accordingly, this section reviews the related issues and conflicting research results.

COVID-19’s spatial distribution in China was well explained by human mobility data in the early stages of the pandemic (until February 10, 2020) outside of Wuhan, China (Kraemer et al. 2020). However, after control measures were implemented, this correlation dropped and pandemic growth rates became negative in most China locations. The authors asserted that travel restrictions may have effectively reduced the flow of case importations from Wuhan in the early stages of the pandemic. However, restrictions may have been less effective once the outbreak was more widespread, thus other local mitigation measures may have been more important to mitigating spread. Another study using data from China also found that the fastest and most widespread way to prevent the spread of COVID-19 infection is to control the route connected to the epicenter in the early stages of the epidemic (Lu et al. 2021). If the virus is widespread, implementing restrictions in hub cities is much more efficient than imposing the same travel restriction across the country (Lu et al. 2021). Another interesting simulation determined when mobility restrictions effectively reduced the pandemic’s size within and between heterogeneous neighboring communities, including one with a high infection risk and another with a low infection risk (Espinoza et al. 2020). The study found that the number of secondary cases increased with the level of mobility, increasing the overall final pandemic magnitude. However, the cordon sanitaire did not always minimize the overall number of infected individuals. Accordingly, the authors argued that mobility restrictions may not always effectively contain disease spread that is evaluated by overall final pandemic size.

A few studies compared the effectiveness of various measurements. Martin-Calvo et al. (2020) used a SEIR model to evaluate the impact of different social distancing strategies under various what-if-scenarios for control and mitigation in Boston, US. The results showed that passive social distance strategies were not enough to contain the pandemic, while active strategies (i.e., large scale testing, remote symptom monitoring, isolation, and contact tracing) are needed. In addition, full confinement was not feasible and did not solve the problem without active measures in place after confinement in case a new outbreak occurred. Askitas et al. (2020) conducted a similar study that examined the impact of various non-pharmaceutical interventions on COVID-19 incidence and mobility patterns for 135 countries. The findings showed that canceling public events and restricting gatherings had the largest effects on limiting the pandemic. Workplace and school closures and stay-at-home requirements also had an effect, but it was not as large. Conversely, internal movement restrictions, public transport closures, and international travel controls did not have a significant impact on reducing new infections.

City lockdowns and travel bans are also controversial and do not always successfully control COVID-19 infections. Based on the susceptible-infection-recovery (SIR) model, Zhang et al. (2020a) argued that lockdown measures, for example those adopted by China, have a severe social-economic cost and may not be a feasible solution for other countries, because there was no strong connection between population flow and cross-regional infection except at the very early stage of the outbreak. The authors asserted that non-lockdown-type measures may have outcomes similar to lockdowns if the measures are quickly prepared and strictly executed. Muller et al. (2020) also claimed that a single restriction strategy (i.e., a complete removal of infections in childcare, primary schools, or workplaces) is not sufficient to control infection dynamics. In addition, the estimated delay of contagion was smaller than expected depending on the model scenarios (Anzai et al. 2020). Therefore, even if the results show positive effects, some measures do not work everywhere (Arellana et al. 2020).

Two interesting studies discussed unexpected effects of social distancing. US state and local government interventions decreased daily mobility by between 45% and 55% as of late April 2020, and person-to-person contact events decreased further by 65–75% on average (Klein et al. 2020a). However, after social distancing guidelines expired on April 30, 2020, mobility and contact patterns increased slightly by 14% as of early May 2020. Ghader et al. (2020) observed a similar trend when examining COVID-19 and social distancing policy effects on human mobility in the US. They found that when COVID-19 cases first emerged (i.e., early- to mid-March, 2020), social distancing statistics (i.e., percentage staying home, number of trips per person, trip distance, etc.) began to improve, regardless of government social distancing orders. However, these statistics stopped improving after about two weeks, despite continuously increased COVID-19 cases and government stay-at-home orders. The authors called this unexpected mobility and COVID-19 case trend “social distancing inertia.” This phenomenon was universal throughout US states, despite different COVID-19 case timelines and government orders in each state. The authors concluded that: (1) those able to follow social distancing orders had already done so before government intervention was adopted, and (2) there is a natural behavior inertia on social distancing, which limits improvement related to social distancing (Ghader et al. 2020).

Although many governments discouraged public transit to limit COVID-19 spread, whether public transport actually spreads the virus is another debate, because there is currently a lack of comprehensive research or scientific evidence on that (Liu et al. 2022; Zhang et al. 2021c; Bucsky 2020; Wielechowski et al. 2020) found a negative but insignificant relationship between human mobility changes in public transport and the number of confirmed COVID-19 cases in Poland, although the strength and statistical significance of the correlation varied substantially across regions. However, there was a strong, negative, and significant correlation between public transport mobility changes and the stringency of government anti-COVID-19 policies. Therefore, the authors argued that forced lockdowns effectively enforced social distancing in public transport, and government travel restrictions contributed to decreased mobility. However, Borsati et al. (2022); Schwartz (2020b) concluded that there is no direct correlation between urban public transit ridership and excess mortality or COVID-19 transmission. When comparing 418 policy measures from six developed countries (Australia, Canada, Japan, New Zealand, the UK, and the US Zhang et al. (2021c) also found that none of the measures in public health and transport is associated with a reduction of either cumulative deaths or cumulative infection cases. Based on US case studies, Schwartz (2020b) emphasized that COVID-19 cases are primarily associated with local community spread, rather than public transit ridership rates. Furthermore, Musselwhite et al. (2020) argued that, although infectious diseases spread in dense public transport vehicles, this does not support the effectiveness of restricting public transport services to prevent spread. As with the influenza case, infection in the subway is very rare (Cooley et al. 2011), while the risk of infection within household contact may be greater (Williams et al. 2010).

## Discussion on future research perspectives

Individuals and governments have worked to reduce the spread of COVID-19. Although it is difficult to pinpoint the transportation sector’s role and influence, it is necessary to analyze the various COVID-19 related factors in more detail to extract the direct influence of travel behavior changes. This chapter reviews the limitations of previous studies (especially the lack of data) and the need for future research directions related thereto and presents topics that require further research in the future.

While extensive studies presented comparative research using revealed data, most were aggregated data forms or surveys. Large scale aggregated data, obtained from Google and Apple mobility reports and mobile devices, are easy to use and great information sources for identifying overall mobility trends under different government measures. However, such aggregated data are not random, represent a small group of individuals, and cannot explain the exact population behavior or capture the interpersonal contacts (Wen et al. 2021; Wellenius et al. 2021; Moslem et al. 2020). In addition, aggregated data cannot capture physical proximity to other people (Dasgupta et al. 2020). To avoid sample-related biases, large scale surveys using rapid data collection technologies are needed (Pawar et al. 2020).

The absence of strong mobility correlations and evidence coupled with studies’ reliance on aggregated or sample data suggests a need for different approaches, for example, analysis of individual level data. Given that COVID-19 is spread through person-to-person contact and the impact may vary depending on individuals’ movements, demographic and socioeconomic characteristics, travel frequencies, activity frequency and locations, and health status (Shi and Fang 2020; Arellana et al. 2020; Chen, 2020). Microscopic analyses could be conducted with individual travel trajectory, POI, and GPS location data (Nian et al. 2020). Another approach involves developing an agent-based simulation to identify individual movements, incorporate contact tracing information, and examine individual infection potential (Heiler et al. 2020; Pan et al. 2020). However, technical challenges (i.e., location uncertainty or spatial error) and sampling bias still need to be addressed to improve accuracy (Gao et al. 2020c).

While many researchers agree on some heterogeneity between COVID-19 prevalence and travel behavior, they emphasize the need for detailed spatiotemporal studies in different cities. Some studies analyzed spatial and temporal correlations, but analyses were limited by a lack of data (Zhao et al., 2020a). Analyses on short- and long-term COVID-19 impacts on travel behavior and overall mobility are lacking (Heiler et al. 2020). In addition, few studies have considered post pandemic factors, especially since it is difficult to predict exactly when the pandemic will end. Most data and studies reviewed in this study were likely conducted in the early phase of the outbreak and may not represent whole waves; future studies should include more detailed and longitudinal studies that examine COVID-19 evolution over a longer period and measure how expectations, experience, activity, and travel behaviors change over time, both during the pandemic and after it ends (De Haas et al. 2020; Pan et al. 2020).

A more complex and intensive application that employs the most advanced technologies combined with qualitative analysis can provide a deeper understanding of travel behavior in response to COVID-19, limit continued virus spread, and provide information for developing adequate prevention policies to manage future diseases with pandemic potential. For a more thorough analysis of the spatiotemporal effectiveness and efficiency of the government’s COVID-19 measures related to travel restrictions, additional studies needed in the future are:

- investigating changes in long-term and short-term travel behavior according to stages of the spread of COVID-19 and government measures;
- extensive investigation of controversial issues (i.e., effects of travel restriction policies on virus infection);
- in particular, regarding the estimation of infection risk levels in public transport, new evidence, and a thorough comparative analysis of previous research methodologies;
- analysis of how the analysis methodology of existing studies can affect the results;
- examining social distancing regulations’ implications for public transit (i.e., capacity or occupancy levels, a solution for expanding passenger demand);
- analyzing different public transit funding mechanisms, such as optimal transit fare structures;
- modeling a wide range of scenarios for shared mobility, paratransit modes, ride-sharing, ride-hailing, and carpooling;
- assessing alternative transportation services in rural and low-income areas; evaluating social equity issues related to transport availability;
- analyzing urban environment effects (i.e., land use and density) associated with travel patterns;
- exploring geographic heterogeneity, such as comparing urban and rural areas and international experiences;
- examining travel information’s effect on mitigating public transportation crowding; and.
- developing more sophisticated interactive simulation platforms that use real time data and provide simulation outputs with adequate indicators under various scenarios.

## Conclusions

The unprecedented COVID-19 outbreak significantly influenced nearly every aspect of daily life across the globe, resulting in dramatic changes in human mobility and activities, and leading to a preference for cars and active modes over public transport. Prevention strategies and travel related control policies also significantly affected urban mobility. Considering the pandemic’s magnitude and severity, both researchers and policy makers need to understand how people respond to the virus and to government restriction policies to reduce the potential for disease spread.

This study reviewed extensive evidence from many countries to investigate the relationship between human mobility and COVID-19 spread, COVID-19’s impact on mobility, and the effect of government countermeasures to reduce mobility to limit disease spread. Findings from this review are summarized as follows:

- Although many studies investigated the effectiveness of government measures to limit mobility, controversy remains regarding whether a single restriction measure can effectively reduce COVID-19 cases.
- City lockdowns and strict travel restrictions carry severe social-economic costs and may not be a feasible solution in some countries. No strong evidence emerged supporting a consistent connection between population flow and cross-regional infection except in a very early stage of the outbreak.
- Government measures regarding social distancing in response to COVID-19 seemed to be effective, but there was not strong evidence supporting a strict limit on human movement itself.
- Research in several countries showed that social distancing effectively reduced COVID-19 spread. There is general agreement that COVID-19 spreads through social activities in specific places (i.e., workplaces) and social gatherings after travel.
- Preparing for a “new normal” after the pandemic is recommended. The pandemic’s long-term consequences may lead to a new era involving economic and social changes, such as smart working and other daily activity patterns, that may reduce future mobility needs.

## Electronic supplementary material

Below is the link to the electronic supplementary material.

Supplementary Material 1

Supplementary Material 2

## References

[CR3] Abdullah, M., Dias, C., Muley, D., Shahin, M.: Exploring the Impacts of COVID-19 on Travel Behavior and mode Preferences, p. 8. Transportation Research Interdisciplinary Perspectives (2020) 10.1016/j.trip.2020.100255 PMC7640923 34173481

[CR2] Abdullah, M., Ali, N., Javid, M.A., Dias, C., Campisi, T.: Public transport versus solo travel mode choices during the COVID-19 pandemic: Self-reported evidence from a developing country.Transportation Engineering, 5. (2021)

[CR1] Abdullah M, Ali N, Aslam AB, Javid MA, Hussain SA (2022). Factors affecting the mode choice behavior before and during COVID-19 pandemic in Pakistan. Int. J. Transp. Sci. Technol.

[CR4] Abu-Rayash, A., Dincer, I.: Analysis of Mobility Trends During the COVID-19 Coronavirus Pandemic: Exploring the Impacts on Global Aviation and Travel in Selected Cities, p. 68. Energy Research & Social Science (2020) 10.1016/j.erss.2020.101693 PMC7365059 32839706

[CR5] Advani M, Sharma N, Dhyani R (2021). Mobility change in Delhi due to COVID and its’ immediate and long term impact on demand with intervened non-motorized transport friendly infrastructural policies. Transp. Policy.

[CR6] Aghabayk K, Esmailpour J, Shiwakoti N (2021). Effects of COVID-19 on rail passengers’ crowding perceptions. Transp. Res. Part A.

[CR7] Ahangari, S., Chavis, C., Jeihani, M.: Public transit ridership analysis during the COVID-19 pandemic. MedRxiv preprint. Available at (2020). 10.1101/2020.10.25.20219105 (accessed on 3 February, 2021)

[CR8] Airak, S., Sukor, N.S.A., Rahman, N.A.: Travel behaviour changes and risk perception during COVID-19: A case study of Malaysia.Transportation Research Interdisciplinary Perspectives,18. (2023) 10.1016/j.trip.2023.100784 PMC9939401 36844954

[CR9] Alama MJ, Shahriera H, Anika MAH, Habib MA (2022). Activity-based integrated modelling for assessing COVID-19 impacts on transport operations and emissions. Transp. Lett.

[CR10] Alidadi, M., Sharifi, A.: Effects of the built environment and human factors on the spread of COVID-19: A systematic literature review.Science of the Total Environment,850. (2022) 10.1016/j.scitotenv.2022.158056 PMC9383943 35985590

[CR11] Almlof, E., Rubensson, I., Cebecauer, M., Jenelius, E.: Who continued traveling by public transport during COVID-19? Socioeconomic factors explaining travel behaviour in Stockholm 2020 based on smart-card data. European Transport Research Review, 13(31). (2021) 10.1186/s12544-021-00488-0 PMC8180438 38624666

[CR12] Aloi, A., Alonso, B., Benavente, J., Cordera, R., Echaniz, E., Gonzalez, F., Ladisa, C., Lezama-Romanelli, R., Lopez-Parra, A., Mazzei, V., Perrucci, L., Prieto-Quintana, D., Rodriguez, A., Sanudo, R.: Effects of the COVID-19 lockdown on urban mobility: Empirical evidence from the city of Santander (Spain).Sustainability, (2020). 12(9).

[CR13] Anzai A, Kobayashi T, Linton NM, Kinoshita R, Hayashi K, Suzuki A, Yang Y, Jung S, Miyama T, Akhmetzhanov AR, Nishiura H (2020). Assessing the impact of reduced travel on exportation dynamics of novel coronavirus infection (COVID-19). J. Clin. Med.

[CR14] Arellana, J., Marquez, L., Cantillo, V.: COVID-19 outbreak in Colombia: An analysis of its impacts on transport systems. Journal of Advanced Transportation, 2020. (2020)

[CR15] Arimura, M., Ha, T.V., Okumura, K., Asada, T.: Changes in urban mobility in Sapporo city, Japan due to the Covid-19 emergency declarations.Transportation Research Interdisciplinary Perspectives,7. (2020) 10.1016/j.trip.2020.100212 PMC7834002 34173468

[CR16] Askitas, N., Tatsiramos, K., Verheyden: Lockdown strategies, mobility patterns and COVID-19. Discussion Paper Series, IZA DP No. 13293, Institute of Labor Economics. (2020)

[CR17] Astroza, S., Tirachini, A., Hurtubia, R., Carrasco, J.A., Guevara, A., Munizaga, M., Figueroa, M., Torres, V.: Mobility changes, teleworking, and remote communication during the COVID-19 Pandemic in Chile. Transport Findings. Available at (2020). 10.32866/001c.13489 (accessed on 1 February, 2021)

[CR18] Awad-Núñez, S., Julio, R., Gomez, J., Moya-Gómez, B., González, J.S.: Post-COVID-19 travel behaviour patterns: impact on the willingness to pay of users of public transport and shared mobility services in Spain.European Transport Research Review,13. (2021) 10.1186/s12544-021-00476-4 PMC7944722 38624604

[CR19] Badr HS, Du H, Marshall M, Dong E, Squire MM, Gardner LM (2020). Association between mobility patterns and COVID-19 transmission in the USA: A mathematical modelling study. Lancet Infect. Dis.

[CR21] Balbontin, C., Hensher, D.A., Beck, M.J., Giesen, R., Basnak, P., Vallejo-Borda, J.A., Venter, C.: Impact of COVID-19 on the number of days working from home and commuting travel: A cross-cultural comparison between Australia, South America and South Africa.Journal of Transport Geography,96. (2021) 10.1016/j.jtrangeo.2021.103188 PMC8413364 34493910

[CR20] Balbontin, C., Hensher, D.A., Beck, M.J.: Advanced modelling of commuter choice model and work from home during COVID-19 restrictions in Australia.Transportation Research Part E,162. (2022) 10.1016/j.tre.2022.102718 PMC9040400 35497404

[CR22] Basnak P, Giesen R, Mu˜noz JC (2022). Estimation of crowding factors for public transport during the COVID-19 pandemic in Santiago, Chile. Transp. Res. Part A.

[CR23] Beck MJ, Hensher DA (2020). Insights into the impact of COVID-19 on household travel and activities in Australia – The early days of easing restrictions. Transp. Policy.

[CR24] Beck MJ, Hensher DA (2020). Insights into the impact of COVID-19 on household travel and activities in Australia – The early days under restrictions. Transp. Policy.

[CR25] Benita, F.: Human Mobility Behavior in COVID-19: A Systematic Literature Review and Bibliometric Analysis, p. 70. Sustainable Cities and Society (2021) 10.1016/j.scs.2021.102916 PMC9187318 35720981

[CR26] Bernardes, S.D., Bian, Z., Thambiran, S.S.M., Gao, J., Na, C., Zuo, F., Hudanich, N., Bhattacharyya, A., Ozbay, K., Iyer, S., Chow, J.Y.J., Nassif, H.: NYC recovery at a glance – The rise of buses and micromobility. C2 Smart White Paper Issue 4, arXiv preprint arXiv:2009.14019 (2020)

[CR27] Bhaduri, E., Manoj, B.S., Wadud, Z., Goswami, A.K., Ghoudhury, C.F.: Modelling the Effects of COVID-19 on Travel mode Choice Behaviour in India, p. 8. Transportation Research Interdisciplinary Perspectives (2020)

[CR29] Borkowski, P., Jazdzewska-Gutta, M., Szmelter-Jarosz, A.: Lockdowned: Everyday mobility changes in response to COVID-19.Journal of Transport Geography,90. (2021) 10.1016/j.jtrangeo.2020.102906 PMC9188832 35721765

[CR30] Borsati M, Nocera S, Percoco M (2022). Questioning the spatial association between the initial spread of COVID-19 and transit usage in Italy. Res. Transp. Econ.

[CR31] Bounie, D., Camara, Y., Galbraith, J.W.: Consumers’ mobility, expenditure and online-offline substitution response to COVID-19: Evidence from French transaction data. Available at (2020). https://ssrn.com/abstract=3588373 (Accessed on 30 March, 2021)

[CR32] Bucsky, P.: Modal Share Changes due to COVID-19: The case of Budapest, p. 8. Transportation Research Interdisciplinary Perspectives (2020) 10.1016/j.trip.2020.100141 PMC7290209 34171021

[CR34] Buhat, C.A.H., Lutero, D.S.M., Olave, Y.H., Torres, M.C., Rabajante, J.F.: Modeling the transmission of respiratory infectious diseases in mass transportation systems. Available at (2020). 10.1101/2020.06.09.20126334 (accessed on 12 January, 2021)

[CR35] Campisi, T., Basbas, S., Skoufas, A., Akgun, N., Ticali, D., Tesoriere, G.: The impact of COVID-19 pandemic on the resilience of sustainable mobility in Sicily.Sustainability, 12(21). (2020)

[CR36] Carteni, A., Francesco, L.D., Martino, M.: How Mobility Habits Influenced the Spread of the COVID-19 Pandemic: Results from the Italian case Study, p. 741. Science of the Total Environment (2020) 10.1016/j.scitotenv.2020.140489 PMC7313484 32599395

[CR37] Carteni, A., Francesco, L.D., Martino, M.: The role of transport accessibility within the spread of the Coronavirus pandemic in Italy.Safety Science,133. (2021) 10.1016/j.ssci.2020.104999 PMC7489889 32952302

[CR38] Chan, H.F., Skali, A., Savage, D.A., Stadelmann, D., Torgler, B.: Risk attitudes and human mobility during the COVID-19 pandemic.Scientific Reports,10. (2020) 10.1038/s41598-020-76763-2 PMC7669857 33199737

[CR40] Chen, Q., Pan, S.: Transport-related Experiences in China in Response to the Coronavirus (COVID-19), p. 8. Transportation Research Interdisciplinary Perspectives (2020) 10.1016/j.trip.2020.100246 PMC7561335 34173480

[CR42] Chen Z, Zhang Q, Lu Y, Guo Z, Zhang X, Zhang W, Guo C, Liao C, Li Q, Han X, Lu J (2020). Distribution of the COVID-19 epidemic and correlation with population emigration from Wuhan, China. Chin. Med. J.

[CR39] Chen C, Feng T, Gu X, Yao B (2022). Investigating the effectiveness of COVID-19 pandemic countermeasures on the use of public transport: A case study of the Netherlands. Transp. Policy.

[CR41] Chen, Y., Sun, X., Deveci, M., Coffman, D.M.: The impact of the COVID-19 pandemic on the behaviour of bike sharing users.Sustainable Cities and Society,84. (2022b) 10.1016/j.scs.2022.104003 PMC9212929 35756367

[CR43] Chinazzi M, Davis JT, Ajelli M, Gioannini C, Litvinova M, Merler S, Piontti AP, Mu K, Rossi L, Sun K, Viboud C, Xiong X, Yu H, Halloran ME, Longini IM, Vespignani A (2020). The effect of travel restrictions on the spread of the 2019 novel coronavirus (COVID-19) outbreak. Science.

[CR44] Cho SH, Park HC (2021). Exploring the behaviour change of crowding impedance on public transit due to COVID-19 pandemic: Before and after comparison. Transp. Lett.

[CR46] Choi Y, Zou L, Dresner M (2022). The effects of air transport mobility and global connectivity on viral transmission: Lessons learned from Covid-19 and its variants. Transp. Policy.

[CR45] Choi, S.E., Kim, J., Seo, D.: Travel patterns of free-floating e-bike-sharing users before and during COVID-19 pandemic.Cities,132. (2023) 10.1016/j.cities.2022.104065 PMC9606076 36317088

[CR47] Cintia, P., Fadda, D., Giannotti, F., Pappalardo, L., Rossetti, G., Rinzivillo, S., Bonato, P., Fabbri, F., Penone, F., Savarese, M., Checchi, D., Chiaromonte, F., Vineis, P., Guzzetta, G., Riccardo, F., Marziano, V., Poletti, P., Trentini, F., Bella, A., Andrianou, X., Manso, M., Fabiani, M., Bellino, S., Boros, S., Urdiales, A.M., Vescio, M.F., Brusaferro, S., Rezza, G., Pezzotti, P., Ajelli, M., Merler, S.: The relationship between human mobility and viral transmissibility during the COVID-19 epidemics in Italy. (2020). arXiv preprint ArXiv abs/2006.03141.

[CR48] Ciuffini, F., Tengattini, S., Bigazzi, A.Y.: Mitigating increased driving after the COVID-19 pandemic: An analysis on mode share, travel demand, and public transport capacity.Transportation Research Record,1–14. (2021) 10.1177/03611981211037884 PMC10149350 37153203

[CR49] Cooley P, Brown S, Cajka J, Chasteen B, Ganapathi L, Grefenstette J, Hollingsworth CR, Lee BY, Levine B, Wheaton WD, Wagener DK (2011). The role of subway travel in an influenza epidemic: A New York City simulation. J. Urb. Health.

[CR50] Costa, C.S., Pitombo, C.S., Souza, F.L., Ud: Travel behavior before and during the COVID-19 pandemic in Brazil: Mobility changes and transport policies for a sustainable transportation system in the post-pandemic period.Sustainability, 14. (2022)

[CR51] Couture, V., Dingel, J.I., Green, A., Handbury, J., Williams, K.R.: JUE Insight: Measuring movement and social contact with smartphone data: A real-time application to COVID-19.Journal of Urban Economics,127. (2022) 10.1016/j.jue.2021.103328 PMC8886508 35250113

[CR52] Cui, Z., Zhu, M., Wang, S., Wang, P., Zhou, Y., Cao, Q., Kopca, C., Wang, Y.: Traffic performance score for measuring the impact of covid-19 on urban mobility. arXiv preprint arXiv:2007.00648 (2020)

[CR53] Currie G, Jain T, Aston L (2021). Evidence of a post-COVID change in travel behaviour – self-reported expectations of commuting in Melbourne. Transp. Res. Part A.

[CR54] Dahlberg, M., Edin, P.-A., Gronqvist, E., Lyhagen, J., Osth, J., Siretskiy, A., Toger, M.: Effects of the COVID-19 pandemic on population mobility under mild policies: Causal evidence from Sweden. (2020). arXiv preprint arXiv:2004.09087.

[CR55] Das S, Boruah A, Banerjee A, Raoniar R, Nama S, Maurya AK (2021). Impact of COVID-19: A radical modal shift from public to private transport mode. Transp. Policy.

[CR56] Dasgupta, N., Funk, M.J., Lazard, A., White, B.E., Marshall, S.W.: Quantifying the social distancing privilege gap: A longitudinal study of smartphone movement. MedRxiv preprint. Available at (2020). 10.1101/2020.05.03.20084624 (accessed on 19 January, 2021)

[CR57] De Haas, M., Faber, R., Hamersma, M.: How COVID-19 and the Dutch ‘intelligent Lockdown’ Change Activities, work and Travel Behavior: Evidence from Longitudinal data in the Netherlands, p. 6. Transportation Research Interdisciplinary Perspectives (2020) 10.1016/j.trip.2020.100150 PMC7284275 34171019

[CR58] De Vos, J.: The effect of COVID-19 and subsequent social distancing on travel behavior.Transportation Research Interdisciplinary Perspectives, 5. (2020) 10.1016/j.trip.2020.100121 PMC7180344 34171016

[CR59] Dingil AE, Esztergár-Kiss D (2021). The influence of the Covid-19 pandemic on mobility patterns: The first wave’s results. Transp. Lett.

[CR60] Downey L, Fonzone A, Fountas G, Semple T (2022). The impact of COVID-19 on future public transport use in Scotland. Transp. Res. Part A.

[CR61] Echaniz E, Rodríguez A, Cordera R, Benavente J, Alonso B, Sa˜nudo R (2021). Behavioural changes in transport and future repercussions of the COVID-19 outbreak in Spain. Transp. Policy.

[CR62] Ecke, L., Magdolen, M., Chlond, B., Vortisch, P.: How the COVID-19 Pandemic Changes Daily Commuting routines – Insights from the German Mobility Panel, vol. 10, pp. 2175–2182. Case Studies on Transport Policy (2022) 10.1016/j.cstp.2022.10.001 PMC9537114 36245689

[CR63] Espinoza, B., Castillo-Chavez, C., Perrings, C.: Mobility restrictions for the control of epidemics: When do they work?PLoS ONE, 15(7). (2020) 10.1371/journal.pone.0235731 PMC7337314 32628716

[CR64] Falchetta, G., Noussan, M.: The Impact of COVID-19 on transport demand, modal choices, and sectoral energy consumption in Europe. IAEE Energy Forum, May, 2020. (2020)

[CR65] Fang, Y., Nie, Y., Penny, M.: Transmission dynamics of the COVID-19 outbreak and effectiveness of government interventions: A data‐driven analysis.Journal of Medical Virology, 92(6). (2020a) 10.1002/jmv.25750 PMC7228381 32141624

[CR66] Fang, H., Wang, L., Yang, Y.: Human mobility restrictions and the spread of the Novel Coronavirus (2019-nCoV) in China.Journal of Public Economics,191. (2020b) 10.1016/j.jpubeco.2020.104272 PMC7833277 33518827

[CR67] Fatmi MR (2020). COVID-19 impact on urban mobility. J. Urban Manage.

[CR68] Ferreira S, Amorim M, Lobo A, Kern M, Fanderl N, Couto A (2022). Travel mode preferences among german commuters over the course of COVID-19 pandemic. Transp. Policy.

[CR69] Fischedick, M.S., Shan, Y., Hubacek, K.: Implications of COVID-19 lockdowns on surface passenger mobility and related CO2 emission changes in Europe.Applied Energy,300. (2021) 10.1016/j.apenergy.2021.117396 PMC8278838 34305265

[CR70] Galeazzi, A., Cinelli, M., Bonaccorsi, G., Pierri, F., Schmidt, A.L., Scala, A., Pammolli, F., Quattrociocchi, W.: Human Mobility in Response to COVID-19 in France, Italy and UK, p. 11. Scientific Reports (2021) 10.1038/s41598-021-92399-2 PMC8222274 34162933

[CR71] Gao, J., Bernardes, S.D., Bian, Z., Ozbay, K., Iyer, S.: Initial impacts of COVID-19 on transportation systems: A case study of the U.S. epicenter, the New York Metropolitan Area. C2 Smart White Paper, arXiv preprint arXiv:2010.01168 (2020a)

[CR72] Gao, S., Rao, J., Kang, Y., Liang, Y., Kruse, J.: Mapping county-level mobility pattern changes in the United States in response to COVID-19. (2020b). arXiv preprint arXiv:2004.04544

[CR73] Gao, S., Rao, J., Kang, Y., Liang, Y., Kruse, J., Doepfer, D., Sethi, A.K., Reyes, J.F.M., Patz, J., Yandell, B.S.: Mobile phone location data reveal the effect and geographic variation of social distancing on the spread of the COVID-19 epidemic. arXiv preprint arXiv:2004.11430. (2020c)

[CR74] Gao, J., Wang, J., Bian, Z., Bernardes, S.D., Chen, Y., Bhattacharyya, A., Thambiran, S.S.M., Ozbay, K., Iyer, S., Ban, X.J.: The effects of the COVID-19 pandemic on transportation systems in New York City and Seattle, USA. C2 Smart White Paper Issue 2, arXiv:2010.01170 (2020d)

[CR75] Ghader, S., Zhao, J., Lee, M., Zhou, W., Zhao, G., Zhang, L.: Observed mobility behavior data reveal “social distancing inertia”. arXiv preprint arXiv:2004.14748 (accessed on 19 January, 2021). (2020)

[CR76] Gkiotsalitis, K., Cats, O.: Public transport planning adaption under the COVID-19 pandemic crisis: Literature review of research needs and directions.Transport Reviews, 41(3). (2021)

[CR77] Gkiotsalitis K, Cats O (2022). Optimal Frequency Setting of Metro Services in the age of COVID-19 Distancing Measures.

[CR78] Glaeser, E.L., Gorback, C., Redding, S.J.: JUE Insight: How much does COVID-19 increase with mobility? Evidence from New York and four other U.S. cities.Journal of Urban Economics,127. (2022) 10.1016/j.jue.2020.103292 PMC7577256 33106711

[CR79] Gonzalez, A.B.R., Wilby, M.R., Díaz, J.J.V., Pozo, R.F.: Characterization of COVID-19’s impact on mobility and short-term prediction of public transport demand in a mid-size city in Spain.Sensors,21. (2021) 10.3390/s21196574 PMC8512832 34640894

[CR80] Gramsch B, Guevara CA, Munizaga M, Schwartz D, Tirachini A (2022). The effect of dynamic lockdowns on public transport demand in times of COVID-19: Evidence from smartcard data. Transp. Policy.

[CR81] Guzman LA, Arellana J, Oviedo D, Aristiz´abal CAM (2021). COVID-19, activity and mobility patterns in Bogot´a. are we ready for a ‘15-minute city’?. Travel Behav. Soc.

[CR82] Habib Y, Xia E, Hashmi SH, Fareed Z (2021). Non-linear spatial linkage between COVID-19 pandemic and mobility in ten countries: A lesson for future wave. J. Infect. Public Health.

[CR83] Hadjidemetriou, G.M., Sasidharan, M., Kouyialis, G., Parlikad, A.K.: The Impact of Government Measures and Human Mobility Trend on COVID-19 Related Deaths in the UK, p. 6. Transportation Research Interdisciplinary Perspectives (2020) 10.1016/j.trip.2020.100167 PMC7334915 34173458

[CR84] Harantová, V., Hájnik, A., Kalašová, A., Figlus, T.: The Effect of the COVID-19 Pandemic on Traffic flow Characteristics, Emissions Production and fuel Consumption at a Selected Intersection in Slovakia, vol. 15. Energies (2022)

[CR85] Harrington, D.M., Hadjiconstantinou, M.: Changes in commuting behaviours in response to the COVID-19 pandemic in the UK.Journal of Transport & Health,24. (2022) 10.1016/j.jth.2021.101313 PMC8651520 34900585

[CR86] Hasselwander, M., Tamagusko, T., Bigotte, J.F., Ferreira, A., Mejia, A., Ferranti, E.J.S.: Building back Better: The COVID-19 Pandemic and Transport Policy Implications for a Developing Megacity, p. 69. Sustainable Cities and Society (2021) 10.1016/j.scs.2021.102864 PMC9760281 36568855

[CR87] Heiler, G., Reisch, T., Hurt, J., Forghani, M., Omani, A., Hanbury, A., Karimipour, F.: Country-wide mobility changes observed using mobile phone data during COVID-19 pandemic. (2020). arXiv preprint arXiv:2008.10064

[CR89] Hensher DA, Beck MJ, Balbontin C (2021). What does the quantum of working from home do to the value of commuting time used in transport appraisal?. Transp. Res. Part A.

[CR88] Hensher DA, Balbontin C, Beck MJ, Wei E (2022). The impact of working from home on modal commuting choice response during COVID-19: Implications for two metropolitan areas in Australia. Transp. Res. Part A.

[CR90] Hensher, D.A., Beck, M.J., Balbontin, C.: Working from home 22 months on from the beginning of COVID-19: What have we learned for future provision of transport services?Research in Transportation Economics,98. (2023)

[CR91] Heydari, S., Konstantinoudis, G., Behsoodi, A.W.: Effect of the COVID-19 Pandemic on bike-sharing Demand and hire time: Evidence from Santander Cycles in London, p. 16. PLoS ONE (2021) 10.1371/journal.pone.0260969 PMC8639062 34855914

[CR92] Hintermann, B., Schoeman, B., Molloy, J., Schatzmann, T., Tchervenkov, C., Axhausen, K.W.: The impact of COVID-19 on mobility choices in Switzerland.Transportation Research Part A,169. (2023) 10.1016/j.tra.2023.103582 PMC9841083 36685312

[CR93] Hotle, S., Murray-Tuite, P., Singh, K.: Influenza risk Perception and travel-related Health Protection Behavior in the US: Insights for the Aftermath of the COVID-19 Outbreak, vol. 5. Transportation Research Interdisciplinary Perspectives (2020) 10.1016/j.trip.2020.100127 PMC7211591 34171017

[CR94] Huang Z, Loo BPY, Axhausen KW (2023). Travel behaviour changes under work-from-home (WFH) arrangements during COVID-19. Travel Behav. Soc.

[CR95] Iacus, S.M., Natale, F., Santamaria, C., Spyratos, S., Vespe, M.: Estimating and projecting air passenger traffic during the COVID-19 coronavirus outbreak and its socio-economic impact.Safety Science,129. (2020a) 10.1016/j.ssci.2020.104791 PMC7200368 32377034

[CR96] Iacus SM, Santamaria C, Sermi F, Spyratos S, Tarchi D, Vespe M (2020). Human mobility and COVID-19 initial dynamics. Nonlinear Dyn.

[CR97] Jaekel, B., Muley, D.: Transport Impacts in Germany and State of Qatar: An Assessment During the First wave of COVID-19, p. 13. Transportation Research Interdisciplinary Perspectives (2022) 10.1016/j.trip.2022.100540 PMC8752045 35036908

[CR98] Javadinasr M, Maggasy T, Mohammadi M, Mohammadain K, Rahimi E, Salon D, Conway MW, Pendyala R, Derrible S (2022). The long-term effects of COVID-19 on travel behavior in the United States: A panel study on work from home, mode choice, online shopping, and air travel. Transp. Res. Part F.

[CR99] Jenelius, E., Cebecauer, M.: Impacts of COVID-19 on Public Transport Ridership in Sweden: Analysis of Ticket Validations, Sales and Passenger Counts, p. 8. Transportation Research Interdisciplinary Perspectives (2020) 10.1016/j.trip.2020.100242 PMC7575262 34173478

[CR100] Jia JS, Yuan Y, Xu G, Jia J, Christakis NA (2020). Population flow drives spatio-temporal distribution of COVID-19 in China. Nature.

[CR101] Jiang S, Cai C (2022). Unraveling the dynamic impacts of COVID-19 on metro ridership: An empirical analysis of Beijing and Shanghai, China. Transp. Policy.

[CR102] Jiao J, Azimian A (2021). Exploring the factors affecting travel behaviors during the second phase of the COVID-19 pandemic in the United States. Transp. Lett.

[CR103] Jou, R.-C., Yeh, C.-S., Chen, K.-H.: Travel Behavior Changes after COVID-19 Outbreak in Taiwan. Journal of Advanced Transportation, 2022. (2022)

[CR104] Kalter, M.J.O., Geurs, K.T., Wismans, L.: Post COVID-19 teleworking and car use intentions. evidence from large scale GPS-tracking and survey data in the Netherlands.Transportation Research Interdisciplinary Perspectives, 12. (2021) 10.1016/j.trip.2021.100498 PMC8661099 34909635

[CR105] Kartal, M.T., Depren, O., Depren, S.K.: The Relationship Between Mobility and COVID-19 Pandemic: Daily Evidence from an Emerging Country by Causality Analysis, p. 10. Transportation Research Interdisciplinary Perspectives (2021) 10.1016/j.trip.2021.100366 PMC9940612 36844006

[CR107] Kaufman, S.M., Moss, M.L., Mcguinness, K.B., Cowan, N.R., Rudner, C.E., Olivia, L., Jenee, M., Josh, K., Katherine, R., Rachel, W.: Transportation during Coronavirus in New York City. Rudin Center for Transportation Policy & Management, NYU. Available at (2020). https://wagner.nyu.edu/impact/research/publications/transportation-during-coronavirus-nyc (accessed on 12 August, 2020)

[CR108] Khan KS, Kunz R, Kleijnen J, Antes G (2003). Five steps to conducting a systematic review. J. R. Soc. Med.

[CR109] Kim, K.: Impacts of COVID-19 on Transportation: Summary and Synthesis of Interdisciplinary Research, p. 9. Transportation Research Interdisciplinary Perspectives (2021) 10.1016/j.trip.2021.100305 PMC7813510 33763645

[CR110] Kissler, S.M., Kishore, N., prabhu, M., Goffman, D., Beilin, Y., Landau, R., Gyamfi-Bannerman, C., Bateman, B.T., Snyder, J., Razavi, A.S., Katz, D., Gal, J., Bianco, A., Stone, J., Larremore, D., Buckee, C.O., Grad, Y.H.: Reductions in commuting mobility correlate with geographic differences in SARS-CoV-2 prevalence in New York City.Nature Communications, 11(4674), (2020) 10.1038/s41467-020-18271-5 PMC7494926 32938924

[CR111] Klein, B., LaRock, T., McCabe, S., Torres, L., Friedland, L., Privitera, F., Lake, B., Kraemer, M.U.G., Brownstein, J.S., Lazer, D., Eliassi-Rad, T., Scarpino, S.V., Vespignani, A., Chinazzi, M.: Reshaping a nation: Mobility, commuting, and contact patterns during the COVID-19 outbreak. Available at (2020a). https://www.networkscienceinstitute.org/publications/reshaping-a-nation-mobility-commuting-and-contact-patterns-during-the-covid-19-outbreak (accessed on 17 February, 2021)

[CR112] Klein, B., LaRock, T., McCabe, S., Torres, L., Privitera, F., Lake, B., Kraemer, M.U.G., Brownstein, J.S., Lazer, D., Eliassi-Rad, T., Scarpino, S.V., Chinazzi, M., Vespignani, A.: Assessing changes in commuting and individual mobility in major metropolitan areas in the United States during the COVID-19 outbreak. Available at (2020b). https://www.networkscienceinstitute.org/publications/assessing-changes-in-commuting-and-individual-mobility-in-major-metropolitan-areas-in-the-united-states-during-the-covid-19-outbreak (accessed on 7 January, 2021)

[CR113] Kłos-Adamkiewicz, Z., Gutowski, P.: The Outbreak of COVID-19 Pandemic in Relation to Sense of Safety and Mobility Changes in Public Transport Using the Example of Warsaw, vol. 14. Sustainability (2022)

[CR114] Konecny, V., Brídziková, M., Senko, Å.: Impact of COVID-19 and anti-pandemic Measures on the Sustainability of Demand in Suburban bus Transport. The case of the Slovak Republic, p. 13. Sustainability (2021)

[CR115] Kraemer MUG, Yang C-H, Gutierrez B, Wu C-H, Klein B, Pigott DM, du Plessis L, Faria NR, Li R, Hanage WP, Brownstein JS, Layan M, Vespignani A, Tian H, Dye C, Pybus OG, Scarpino SV (2020). The effect of human mobility and control measures on the COVID-19 epidemic in China. Science.

[CR116] Kubal’ák, S., Kalašová, A., Hájnik, A.: The bike-sharing system in slovakia and the Impact of COVID-19 on this shared mobility service in a selected city.Sustainability, 13. (2021)

[CR117] Lau H, Khosrawipour V, Kocbach P, Mikolajczyk A, Ichii H, Zacharski M, Bania J, Khosrawipour T (2020). The association between international and domestic air traffic and the coronavirus (COVID-19) outbreak. J. Microbiol. Immunol. Infect.

[CR119] Lee H, Park SJ, Lee GR, Kim JE, Lee JH, Jung Y, Nam EW (2020). The relationship between trends in COVID-19 prevalence and traffic levels in South Korea. Int. J. Infect. Dis.

[CR120] Lee, M., Zhao, J., Sun, Q., Pan, Y., Zhou, W., Xiong, C., Zhang, L.: Human mobility trends during the early stage of the COVID-19 pandemic in the United States.PLoS ONE, 15(11). (2020b) 10.1371/journal.pone.0241468 PMC7652287 33166301

[CR118] Lee, S., Ko, E., Jang, K., Kim, S.: Understanding individual-level travel behavior changes due to COVID-19: Trip frequency, trip regularity, and trip distance.Cities,135. (2023) 10.1016/j.cities.2023.104223 PMC9889257 36741336

[CR124] Li, T., Wang, J., Huang, J., Yang, W., Chen, Z.: Exploring the dynamic impacts of COVID-19 on intercity travel in China.Journal of Transport Geography,95. (2021a) 10.1016/j.jtrangeo.2021.103153 PMC9759306 36567951

[CR121] Li H, Zhang Y, Zhu M, Ren G (2021). Impacts of COVID-19 on the usage of public bicycle share in London. Transp. Res. Part A.

[CR122] Li, A., Zhao, P., Haitao, H., Mansourian, A., Axhausen, K.W.: How did micro-mobility Change in Response to COVID-19 Pandemic? A case Study Based on spatial-temporal-semantic Analytics, p. 90. Computers, Environment and Urban Systems (2021c) 10.1016/j.compenvurbsys.2021.101703 PMC8492604 34629583

[CR123] Li, A., Zhao, P., He, H., Axhause, K.W.: Understanding the variations of micro-mobility behavior before and during COVID-19 pandemic period. Transportation Research Board 100th Annual Meeting, Washington, DC., USA. (2021d)

[CR125] Limsawasd C, Athigakunagorn N, Khathawatcharakun P, Boonmee A (2022). Skip-stop strategy patterns optimization to enhance mass transit operation under physical distancing policy due to COVID-19 pandemic outbreak. Transp. Policy.

[CR126] Linka K, Peirlinck M, Costabal FS, Kuhl E (2020). Outbreak dynamics of COVID-19 in Europe and the effect of travel restrictions. Comput. Methods Biomech. BioMed. Eng.

[CR131] Liu S, Yamamoto T (2022). Role of stay-at-home requests and travel restrictions in preventing the spread of COVID-19 in Japan. Transp. Res. Part A.

[CR127] Liu K, Ai S, Song S, Zhu G, Tian F, Li H, Gao Y, Wu Y, Zhang S, Shao Z, Liu Q, Lin H (2020). Population movement, city closure in Wuhan, and geographical expansion of the COVID-19 infection in China in January 2020. Clin. Infect. Dis.

[CR128] Liu, H., Bai, X., Shen, H., Pang, X., Liang, Z., Liu, Y.: Synchronized travel restrictions across cities can be effective in COVID-19 control. MedRxiv preprint. Available at (2020b). 10.1101/2020.04.02.20050781 (accessed on 16 February, 2021)

[CR130] Liu, L., Miller, H.J., Scheff, J.: The impacts of COVID-19 pandemic on public transit demand in the United States. PLos One, 15(11), (2020c). 10.1371/journal.pone.0242476 (accessed on 16 March, 2021) 10.1371/journal.pone.0242476 PMC7673535 33206721

[CR129] Liu, X., Kortoçi, P., Motlagh, N.H., Nurmi, P., Tarkoma, S.: A Survey of COVID-19 in Public Transportation: Transmission risk, Mitigation and Prevention, p. 1. Multimodal Transportation (2022)

[CR132] Llaguno-Munitxa, M., Bou-Zeid, E.: Role of vehicular emissions in urban air quality: The COVID-19 lockdown experiment.Transportation Research Part D,115. (2023) 10.1016/j.trd.2022.103580 PMC9771761 36573137

[CR133] Loo, B.P.Y., Huang, Z.: Spatio-temporal Variations of Traffic Congestion Under work from home (WFH) Arrangements: Lessons Learned from COVID-19, p. 124. Cities (2022) 10.1016/j.cities.2022.103610 PMC8784440 35095163

[CR134] Lozzi G, Rodrigues M, Marcucci E, Teoh T, Gatta V, Pacelli V (2020). COVID-19 and Urban Mobility: Impacts and Perspectives. Research for TRAN Committee.

[CR135] Lu, J., Lin, A., Jiang, C., Zhang, A., Yang, Z.: Influence of transportation network on transmission heterogeneity of COVID-19 in China.Transportation Research Part C,129. (2021) 10.1016/j.trc.2021.103231 PMC8169317 34092940

[CR136] Mancinelli, E., Rizza, U., Canestrari, F., Graziani, A., Virgili, S., Passerini, G.: New habits of travellers deriving from COVID-19 pandemic: A survey in ports and airports of the adriatic region.Sustainability, 14. (2022)

[CR137] Manzira, C.K., Charly, A., Caulfield, B.: Assessing the Impact of Mobility on the Incidence of COVID-19 in Dublin City, p. 80. Sustainable Cities and Society (2022) 10.1016/j.scs.2022.103770 PMC8828378 35165649

[CR138] Marra AD, Sun L, Corman F (2022). The impact of COVID-19 pandemic on public transport usage and route choice: Evidences from a long-term tracking study in urban area. Transp. Policy.

[CR139] Mars L, Arroyo R, Ruiz T (2022). Mobility and wellbeing during the covid-19 lockdown. Evidence from Spain. Transp. Res. Part A.

[CR140] Martin-Calvo, D., Aleta, A., Pentland, A., Moreno, Y., Moro, E.: Effectiveness of social distancing strategies for protecting a community from a pandemic with a data driven contact network based on census and real-world mobility data. MIT Connection Science. Available at (2020). https://connection.mit.edu/sites/default/files/publication-pdfs/Preliminary_Report_Effectiveness_of_social_distance_strategies_COVID-19%20(1).pdf (accessed on 16 February, 2021)

[CR141] Mashrur SM, Wang K, Habib KN (2022). Will COVID-19 be the end for the public transit? Investigating the impacts of public health crisis on transit mode choice. Transp. Res. Part A.

[CR142] Medlock, K.B., Temzelides, T., Hung, S.Y.: COVID-19 and the value of safe transport in the United States.Scientific Reports,11. (2021) 10.1038/s41598-021-01202-9 PMC8569113 34737382

[CR143] Meena S (2020). Impact of novel coronavirus (COVID-19) pandemic on travel pattern: A case study of India. Indian J. Sci. Technol.

[CR144] Meister, A., Mondal, A., Asmussen, K.E., Bhat, C., Axhausen, K.W.: Modeling urban mode choice behavior during the COVID-19 pandemic in switzerland using mixed multiple discrete-continuous extreme value models.Transportation Research Record,1–12. (2022)

[CR145] Mogaji, E.: Impact of COVID-19 on Transportation in Lagos, Nigeria, p. 6. Transportation Research Interdisciplinary Perspectives (2020) 10.1016/j.trip.2020.100154 PMC7290214 34171020

[CR146] Mollers A, Specht S, Wessel J (2022). The impact of the Covid-19 pandemic and government intervention on active mobility. Transp. Res. Part A.

[CR147] Morita, H., Nakamura, S., Hayashi, Y.: Changes of urban activities and behaviors due to COVID-19 in Japan. Available at (2020). https://ssrn.com/abstract=3594054 (accessed on 3 February, 2021)

[CR148] Moslem S, Campisi T, Szmelter-Jarosz A, Duleba S, Nahiduzzaman KM, Tesoriere G (2020). Best-worst method for modelling mobility choice after COVID-19: Evidence from Italy. Sustainability.

[CR149] Mouratidis K, Peters S (2022). COVID-19 impact on teleactivities: Role of built environment and implications for mobility. Transp. Res. Part A.

[CR150] Muley D, Shahin M, Dias C, Abdullah M (2020). Role of transport during outbreak of infectious diseases: Evidence from the past. Sustainability.

[CR151] Muller, S.A., Balmer, M., Neumann, A., Nagel, K.: Mobility traces and spreading of COVID-19. MedRxiv preprint. Available at https://doi.org/ (2020). 10.1101/2020.03.27.20045302 (accessed on 25 January, 2021)

[CR152] Musselwhite, C., Avineri, E., Susilo, Y.: Editorial JTH 16 – The Coronavirus disease COVID-19 and implications for transport and health.Journal of Transport & Health,16. (2020) 10.1016/j.jth.2020.100853 PMC7174824 32337154

[CR153] Mussone, L., Changizi, F.: A Study on the Factors that Influenced the Choice of Transport mode Before, During, and After the First Lockdown in Milan, Italy, p. 136. Cities (2023) 10.1016/j.cities.2023.104251 PMC9987251 36911882

[CR154] Navarrete-Hernandez P, Rennert L, Balducci A (2023). An evaluation of the impact of COVID-19 safety measures in public transit spaces on riders’ worry of virus contraction. Transp. Policy.

[CR155] Nian, G., Peng, B., Sun, D., Ma, W., Peng, B., Huang, T.: Impact of COVID-19 on urban mobility during post-epidemic period in Megacities: From the perspectives of taxi travel and social vitality. Sustainability, 12(19). (2020)

[CR156] Nikiforiadis, A., Mitropoulos, L., Kopelias, P., Basbas, S., Stamatiadis, N., Kroustali, S.: Exploring mobility pattern changes between before, during and after COVID-19 lockdown periods for young adults.Cities,125. (2022) 10.1016/j.cities.2022.103662 PMC8923996 35309857

[CR157] Nikolaidou, A., Kopsacheilis, A., Georgiadis, G., Noutsias, T., Politis, I., Fyrogenis: Factors affecting public transport performance due to the COVID-19 outbreak: A worldwide analysis.Cities,134. (2023) 10.1016/j.cities.2023.104206 PMC9841081 36683673

[CR158] Oestreich L, Rhoden PS, Vieira JS, Ruiz-Padillo A (2023). Impacts of the COVID-19 pandemic on the profile and preferences of urban mobility in Brazil: Challenges and opportunities. Travel Behav. Soc.

[CR159] Orro, A., Novales, M., Monteagudo, A., Perez-Lopez, J.-B., Bugarin, M.R.: Impact on city bus transit services of the COVID–19 lockdown and return to the New Normal: The case of a Coruña (Spain).Sustainability, 12(17). (2020)

[CR160] Oum TH, Wang K (2020). Socially optimal lockdown and travel restrictions for fighting communicable virus including COVID-19. Transp. Policy.

[CR161] Oztig LI, Askin OE (2020). Human mobility and coronavirus disease 2019 (COVID-19): A negative binomial regression analysis. Public. Health.

[CR163] Pan Y, He SY (2022). Analyzing COVID-19’s impact on the travel mobility of various social groups in China’s Greater Bay Area via mobile phone big data. Transp. Res. Part A.

[CR162] Pan, Y., Darzi, A., Kabiri, A., Zhao, G., Luo, W., Xiong, C., Zhang, L.: Quantifying human mobility behaviour changes during the COVID-19 outbreak in the United States.Scientific Reports,10. (2020) 10.1038/s41598-020-77751-2 PMC7691347 33244071

[CR164] Pang J, He Y, Shen S (2023). High-speed railways and the spread of Covid-19. Travel Behav. Soc.

[CR165] Parady, G., Taniguchi, A., Takami, K.: Travel behavior changes during the COVID-19 pandemic in Japan: Analyzing the effects of risk perception and social influence on going-out self-restriction.Transportation Research Interdisciplinary Perspectives,7. (2020) 10.1016/j.trip.2022.100649 PMC9239539 35782787

[CR166] Park, J.: Changes in subway ridership in response to COVID-19 in Seoul, South Korea: Implications for social distancing.Cureus, 12(4). (2020) 10.7759/cureus.7668 PMC7163336 32313784

[CR167] Parr, S., Wolshon, B., Renne, J., Murray-Tuite, P., Kim, K.: Traffic impacts of the COVID-19 pandemic: Statewide analysis of social separation and activity restriction.Natural Hazards Review, 21(3). (2020)

[CR168] Pawar, D.S., Yadav, A.K., Akolekar, N., Velaga, N.R.: Impact of physical distancing due to novel coronavirus (SARS-CoV-2) on daily travel for work during transition to lockdown.Transportation Research Interdisciplinary Perspectives,7. (2020) 10.1016/j.trip.2020.100203 PMC7437436 34173467

[CR169] Peng, Y., Lopez, J.M.R., Santos, A.P., Mobeen, M., Scheffran, J.: Simulating exposure-related human mobility behavior at the neighborhood-level under COVID-19 in Porto Alegre, Brazil, Cities, 134. (2023) 10.1016/j.cities.2022.104161 PMC9800815 36597474

[CR170] Pepe, E., Bajardi, P., Gauvin, L., Privitera, F., Lake, B., Cattuto, C., Tizzoni, M.: COVID-19 outbreak response, a dataset to assess mobility changes in Italy following national lockdown.Scientific Data, 7(230). (2020) 10.1038/s41597-020-00575-2 PMC7343837 32641758

[CR171] Peralvo, F.C., Vanegas, P.C., Ord´o˜nez, E.A.: A systematic review of COVID-19 transport policies and mitigation strategies around the globe.Transportation Research Interdisciplinary Perspectives,15. (2022) 10.1016/j.trip.2022.100653 PMC9289094 35873107

[CR172] Pozo, R.F., Wilby, M.R., Díaz, J.J.V., Gonz´alez, A.B.R.: Data-driven analysis of the impact of COVID-19 on Madrid’s public transport during each phase of the pandemic.Cities,127. (2022) 10.1016/j.cities.2022.103723 PMC9057950 35530724

[CR173] Przybylowski, A., Stelmak, S., Suchanek, M.: Mobility behaviour in view of the impact of the COVID-19 pandemic – Public transport users in Gdansk case study.Sustainability, 13(1). (2021)

[CR175] Pullano, G., Valdano, E., Scarpa, N., Rubrichi, S., Colizza, V.: Population mobility reductions during COVID-19 epidemic in France under lockdown. MedRxiv preprint. Available at (2020). 10.1101/2020.05.29.20097097 (accessed on 8 January, 2021)

[CR176] Rasca, S., Markvica, K., Ivanschitz, B.P.: Impacts of COVID-19 and Pandemic Control Measures on Public Transport Ridership in European Urban areas – The Cases of Vienna, Innsbruck, Oslo, and Agder, p. 10. Transportation Research Interdisciplinary Perspectives (2021) 10.1016/j.trip.2021.100376 PMC8422269 34514371

[CR177] Rosik P, Komornicki T, Duma P, Goliszek S (2022). The effect of border closure on road potential accessibility in the regions of the EU-27. The case of the COVID-19 pandemic. Transp. Policy.

[CR178] Rothengatter W, Zhang J, Hayashi Y, Nosach A, Wang K, Oum TH (2021). Pandemic waves and the time after Covid-19 – consequences for the transport sector. Transp. Policy.

[CR179] Ruiz-Euler, A., Privitera, F., Giuffrida, D., Lake, B., Zara, I.: Mobility patterns and income distribution in times of crisis: U.S. urban centers during the COVID-19 Pandemic. Available at (2020). https://ssrn.com/abstract=3572324 (accessed on 14 January, 2021)

[CR180] Saladie, O., Bustamante, E., Gutierrez, A.: COVID-19 Lockdown and Reduction of Traffic Accidents in Tarragona Province, Spain, p. 8. Transportation Research Interdisciplinary Perspectives (2020) 10.1016/j.trip.2020.100218 PMC7474913 34173472

[CR181] Sangveraphunsiri T, Fukushige T, Jongwiriyanurak N, Tanaksaranond G, Jarumaneeroj P (2022). Impacts of the COVID-19 Pandemic on the spatio-temporal Characteristics of a bicycle-sharing System: A case Study of Pun Pun.

[CR182] Santamaria, C., Sermi, F., Spyratos, S., Iacus, S.M., Annunziato, A., Tarchi, D., Vespe, M.: Measuring the impact of COVID-19 confinement measures on human mobility using mobile positioning data: A European regional analysis.Safety Science,132. (2020) 10.1016/j.ssci.2020.104925 PMC7486861 32952303

[CR183] Sasidharan, M., Singh, A., Torbaghan, M.E., Parlikad, A.K.: A vulnerability-based Approach to human-mobility Reduction for Countering COVID-19 Transmission in London While Considering Local air Quality, p. 741. Science of the Total Environment (2020) 10.1016/j.scitotenv.2020.140515 PMC7315141 32887014

[CR184] Schaefer KJ, Tuitjer L, Keitel ML (2021). Transport disrupted – substituting public transport by bike or car under Covid 19. Transp. Res. Part A.

[CR185] Schlosser, F., Maier, B.F., Jack, O., Hinrichs, D., Zachariae, A., Brockmann, D.: COVID-19 lockdown induces disease-mitigating structural changes in mobility networks.PNAS, Proceedings of the National Academy of Sciences of the United States of America, 117(52). (2020) 10.1073/pnas.2012326117 PMC7776901 33273120

[CR186] Schwartz, S.: Global mobility response to COVID-19: How cities are responding, recovering, and reopening transportation systems around the world. Available at (2020a). https://www.samschwartz.com/staff-reflections/2020/6/globalresponse (accessed on 6 October, 2020)

[CR187] Schwartz S (2020). Public Transit and COVID-19 Pandemic: Global Research and best Practices.

[CR188] Shaheen, S., Wong, S.: Public transit and shared mobility COVID-19 recovery: Policy recommendations and research needs. University of California. Available at (2020). https://escholarship.org/uc/item/9nh6w2gq (accessed on 13 January, 2021)

[CR189] Shakibaei, S., de Jong, G.C., Alpkokin, P., Rashidi, T.H.: Impact of the COVID-19 Pandemic on Travel Behavior in Istanbul: A Panel data Analysis, p. 65. Sustainable Cities and Society (2020) 10.1016/j.scs.2020.102619 PMC7682431 33251093

[CR190] Shamshiripour, A., Rahimi, E., Shabanpour, R., Mohammadian, A.: How is COVID-19 Reshaping activity-travel Behavior? Evidence from a Comprehensive Survey in Chicago, p. 7. Transportation Research Interdisciplinary Perspectives (2020) 10.1016/j.trip.2020.100216 PMC7474875 34173469

[CR191] Shelat S, Cats O, Cranenburgh S (2022). Traveller behaviour in public transport in the early stages of the COVID-19 pandemic in the Netherlands. Transp. Res. Part A.

[CR192] Shi, Z., Fang, Y.: Temporal relationship between outbound traffic from Wuhan and the 2019 coronavirus disease (COVID-19) incidence in China. MedRxiv preprint. Available at (2020). 10.1101/2020.03.15.20034199 (accessed on 22 March, 2021)

[CR193] Simovi´c, S., Ivaniševi´c, T., Bradic´, B., Cˇ icˇevic´, S., Trifunovic´, A.: What causes changes in passenger behavior in South-East Europe during the COVID-19 pandemic? Sustainability,13. (2021)

[CR194] Snyder H (2019). Literature review as a research methodology: An overview and guidelines. J. Bus. Res.

[CR195] Sobieralski, J.B.: COVID-19 and airline employment: Insights from historical uncertainty shocks to the industry.Transportation Research Interdisciplinary Perspectives, 5. (2020) 10.1016/j.trip.2020.100123 PMC7322265 34173453

[CR196] Sogbe E (2021). The evolving impact of coronavirus (COVID-19) pandemic on public transportation in Ghana. Case Stud. Transp. Policy.

[CR197] Sokadjo, Y.M., Atchade, M.N.: The Influence of Passenger air Traffic on the Spread of COVID-19 in the World, p. 8. Transportation Research Interdisciplinary Perspectives (2020) 10.1016/j.trip.2020.100213 PMC7833922 34173471

[CR198] Song, J., Zhang, L., Qin, Z., Ramli, M.A.: Spatiotemporal evolving patterns of bike-share mobility networks and their associations with land-use conditions before and after the COVID-19 outbreak.Physica A,592. (2022) 10.1016/j.physa.2021.126819 PMC8719369 35002051

[CR199] Su, M., Hu, B., Jiang, Y., Zhang, Z., Li, Z.: Relationship between the Chinese main air transport network and COVID-19 pandemic transmission.Mathematics,10. (2022a)

[CR200] Su M, Hu B, Luan W, Tian C (2022). Effects of COVID-19 on China’s civil aviation passenger transport market. Res. Transp. Econ.

[CR201] Suman HK, Agarwal A, Bolia NB (2020). Public transport operations after lockdown: How to make it happen?. Trans. Indian Natl. Acad. Eng.

[CR202] Sung, H.: Non-pharmaceutical interventions and urban vehicle mobility in Seoul during the COVID-19 pandemic.Cities,131. (2022) 10.1016/j.cities.2022.103911 PMC9359518 35966967

[CR203] Sy, K.T.L., Martinez, M.E., Rader, B., White, L.F.: Socioeconomic Disparities in Subway use and COVID-19 Outcomes in New York City. American Journal of Epidemiology (2020) 10.1093/aje/kwaa277 PMC7799254 33372209

[CR204] Szczepanek, W.K., Kruszyna, M.: The Impact of COVID-19 on the Choice of Transport Means in Journeys to work Based on the Selected Example from Poland, vol. 14. Sustainability (2022)

[CR205] Tan L, Ma C (2021). Choice behavior of commuters’ rail transit mode during the COVID-19 pandemic based on logistic model. J. Traffic Transp. Eng. (English Edition).

[CR206] Teixeira, J.F., Lopes, M.: The link Between bike Sharing and Subway use During the COVID-19 Pandemic: The case-study of New York’s Citi Bike, p. 6. Transportation Research Interdisciplinary Perspectives (2020) 10.1016/j.trip.2020.100166 PMC7345406 34173457

[CR207] Teixeira JF, Silva C, Moura, S´a F (2022). The strengths and weaknesses of bike sharing as an alternative mode during disruptive public health crisis: A qualitative analysis on the users’ motivations during COVID-19. Transp. Policy.

[CR208] Tiikkaja, H., Viri, R.: The Effects of COVID-19 Epidemic on Public Transport Ridership and Frequencies. A case Study from Tampere, Finland, p. 10. Transportation Research Interdisciplinary Perspectives (2021) 10.1016/j.trip.2021.100348 PMC9940608 36844005

[CR209] Tirachini A, Cats O (2020). COVID-19 and public transportation: Current assessment, prospects, and research needs. J. Public Transp.

[CR210] Vannoni, M., McKee, M., Semenza, J.C., Bonell, C., Stuckler, D.: Using volunteered geographic information to assess mobility in the early phases of the COVID-19 pandemic: A cross-city time series analysis of 41 cities in 22 countries from March 2nd to 26th 2020.Globalization and Health,16. (2020) 10.1186/s12992-020-00598-9 PMC7509494 32967691

[CR211] Vichiensan, V., Hayashi, Y., Kamnerdsap, S.: COVID-19 countermeasures and passengers’ confidence of urban rail travel in Bangkok.Sustainability, 13. (2021)

[CR212] Vickerman R (2021). Will Covid-19 put the public back in public transport? A UK perspective. Transp. Policy.

[CR214] Wang, D., Zuo, F., Gao, J., He, Y., Bian, Z., Bernardes, S.D., Na, C., Wang, J., Petinos, J., Ozbay, K., Chow, J.Y.J., Iyer, S., Nassif, H., Ban, X.J.: Agent-based simulation model and deep learning techniques to evaluate and predict transportation trends around COVID-19. C2 Smart White Paper Issue 3, https://arxiv.org/abs/2010.09648 (2020)

[CR213] Wang, Y., Wang, Z., Wang, J., Li, M., Wang, S., He, X., Zhou, C.: Evolution and control of the COVID-19 pandemic: A global perspective.Cities,130. (2022) 10.1016/j.cities.2022.103907 PMC9359505 35966443

[CR215] Wei, Y., Wang, J., Song, W., Xiu, C., Ma, L., Pei, T.: Spread of COVID-19 in China: Analysis from a city-based epidemic and mobility model.Cities,110. (2021) 10.1016/j.cities.2020.103010 PMC7598765 33162634

[CR216] Wellenius, G.A., Vispute, S., Espinosa, V., Fabrikant, A., Tsai, T.C., Hennessy, J., Williams, B., Gadepalli, K., Boulanger, A., Pearce, A., Kamath, C., Schlosberg, A., Bendebury, C., Stanton, C., Bavadekar, S., Pluntke, C., Desfontaines, D., Jacobson, B., Armstrong, Z., Gipson, B., Wilson, R., Widdowson, A., Chou, K., Oplinger, A., Shekel, T., Jha, A.K., Gabrilovich, E.: Impacts of social distancing policies on mobility and COVID-19 case growth in the US.Nature Communications,12. (2021) 10.1038/s41467-021-23404-5 PMC8149701 34035295

[CR217] Wen, L., Sheng, M., Sharp, B.: The Impact of COVID-19 on Changes in Community Mobility and Variation in Transport Modes. New Zealand Economic Papers (2021)

[CR218] WHO:. Coronavirus. World Health Organization. Available at (2021). https://www.who.int/health-topics/coronavirus#tab=tab_1 (accessed on 17 March, 2021)

[CR219] Wielechowski M, Czech K, Grzeda L (2020). Decline in mobility: Public transport in Poland in the time of the COVID-19 pandemic. Economies.

[CR220] Wilbur, M., Ayman, A., Ouyang, A., Poon, V., Kabir, R., Vadali, A., Pugliese, P., Freudberg, D., Laszka, A., Dubey, A.: Impact of COVID-19 on public transit accessibility and ridership. arXiv preprint arXiv:2008.02413. (2020) 10.1177/03611981231160531 PMC10107069 38602901

[CR221] Williams, C., Schweiger, B., Diner, G., Gerlach, F., Haaman, F., Krause, G., Nienhaus, A., Buchholz, U.: Seasonal influenza risk in hospital healthcare workers is more strongly associated with household than occupational exposures: results from a prospective cohort study in Berlin, Germany, 2006/07.BMC Infectious Diseases, 10(8). (2010) 10.1186/1471-2334-10-8 PMC2836320 20067628

[CR222] Wolfswinkel JF, Furtmueller E, Wilderom CPM (2013). Using grounded theory as a method for rigorously reviewing literature. Eur. J. Inform. Syst.

[CR223] Xu, P., Dredze, M., Broniatowski, D.A.: The twitter social mobility index: Measuring social distancing practices from geolocated tweets.Journal of Medical Internet Research, 22(12). (2020) 10.2196/21499 PMC7717895 33048823

[CR224] Yabe, T., Tsubouchi, K., Fujiwara, N., Wada, T., Sekimoto, Y., Ukkusuri, S.V.: Non-compulsory measures sufficiently reduced human mobility in Tokyo during the COVID-19 epidemic.Scientific Reports,10. (2020) 10.1038/s41598-020-75033-5 PMC7581808 33093497

[CR225] Yang, S., Chen, Z.: The impact of COVID-19 on high-speed rail and aviation operations.Sustainability, 14. (2022)

[CR226] Yang, Y., Cao, M., Cheng, L., Zhai, K., Zhao, X., Vos, J.D.: Exploring the Relationship Between the COVID-19 Pandemic and Changes in Travel Behaviour: A Qualitative Study, p. 11. Transportation Research Interdisciplinary Perspectives (2021) 10.1016/j.trip.2021.100450 PMC8452907 34568810

[CR227] Yilmazkuday, H.: COVID-19 spread and inter-county travel: Daily evidence from the U.S. Transportation Research Interdisciplinary Perspectives, 8. (2020) 10.1016/j.trip.2020.100244 PMC7580684 34173479

[CR228] Yuksel, M., Aydede, Y., Begolli, F.: Dynamics of social mobility during the COVID-19 pandemic in Canada. Discussion Paper Series, IZA DP No. 13376, Institute of Labor Economics. (2020)

[CR229] Zavareh MF, Mehdizadeh M, Nordfjærn T (2022). Demand for mitigating the risk of COVID-19 infection in public transport: The role of social trust and fatalistic beliefs. Transp. Res. Part F: Psychol. Behav.

[CR232] Zhang, X., Ji, Z., Zheng, Y., Ye, X., Li, D.: Evaluating the effect of city lock-down on controlling COVID-19 propagation through deep learning and network science models.Cities,107. (2020a) 10.1016/j.cities.2020.102869 PMC7402371 32834328

[CR235] Zhang Y, Zhang A, Wang J (2020). Exploring the roles of high-speed train, air and coach services in the spread of COVID-19 in China. Transp. Policy.

[CR230] Zhang, L., Ghader, S., Pack, M.L., Xiong, C., Darzi, A., Yang, M., Sun, Q., Kabiri, A., Hu, S.: An interactive COVID-19 mobility impact and social distancing analysis platform. Transportation Research Board 100th Annual Meeting, Washington, DC., USA. (2021a) 10.1177/03611981211043813 PMC10152223 37153196

[CR231] Zhang J, Hayashi Y, Frank LD (2021). COVID-19 and transport: Findings from a world-wide expert survey. Transp. Policy.

[CR234] Zhang J, Zhang R, Ding H, Li S, Liu R, Ma S, Zhai B, Kashima S, Hayashi Y (2021). Effects of transport-related COVID-19 policy measures: A case study of six developed countries. Transp. Policy.

[CR236] Zhao S, Zhuang Z, Cao P, Ran J, Gao D, Lou Y, Yang L, Cai Y, Wang W, He D, Wang MH (2020). Quantifying the association between domestic travel and the exportation of novel coronavirus (2019-nCoV) cases from Wuhan, China in 2020: A correlational analysis. J. Travel Med.

[CR237] Zhao, S., Zhuang, Z., Ran, J., Lin, J., Yang, G., Yang, L., He, D.: The Association Between Domestic Train Transportation and Novel Coronavirus (2019-nCoV) Outbreak in China from 2019 to 2020: A data-driven Correlational Report, p. 33. Travel Medicine and Infectious Disease (2020b) 10.1016/j.tmaid.2020.101568 PMC7128735 32006656

[CR238] Zheng, R., Xu, Y., Wang, W., Ning, G., Bi, Y.: Spatial Transmission of COVID-19 via Public and Private Transportation in China, p. 34. Travel Medicine and Infectious Disease (2020) 10.1016/j.tmaid.2020.101626 PMC7118651 32184132

[CR239] Zhou K, Hu D, Li F (2022). Impact of COVID-19 on private driving behavior: Evidence from electric vehicle charging data. Transp. Policy.

[CR240] Zhu, P., Guo, Y.: The role of high-speed rail and air Travel in the Spread of COVID-19 in China, p. 42. Travel Medicine and Infectious Disease (2021) 10.1016/j.tmaid.2021.102097 PMC8166159 34082087

[CR241] Zubair, H., Karoonsoontawong, A., Kanitpong, K.: Effects of COVID-19 on Travel Behavior and mode Choice: A case Study for the Bangkok Metropolitan Area, p. 14. Sustainability (2022)

[CR242] Zuo, F., Wang, J., Gao, J., Ozbay, K., Ban, X.J., Shen, Y., Yang, H., Iyer, S.: An interactive data visualization and analytics tool to evaluate mobility and sociability trends during COVID-19. In San Diego ’20: The 9th SIGKDD International Workshop for Urban Computing, August 24, 2020, San Diego, CA. ACM, New York, NY, USA. (2020)

