# Supplementary material for: Systematic literature review on impacts of COVID-19 pandemic and corresponding measures on mobility
Source: Transportation (Amst). 2023 Apr 25:1–55. Online ahead of print. doi: 10.1007/s11116-023-10392-2 (PMC10126540; doi:10.1007/s11116-023-10392-2)
Supplement: Supplementary file 2 — Supplementary Material 2 [file 11116_2023_10392_MOESM2_ESM.docx]

**Responses to Reviewers’ Comments**

As you pointed out, we searched recently published papers (including 2022 through March 2023) and found 52 papers, of which 21 papers that were relevant to this study were added. The related themes of them are specifically as follows.

Airak et al. (2023) and Navarrete-Hernandez et al. (2023) : added citation related to risk perception in section 3.5 (p. 17)

Choi et al. (2023) : added text regarding bike sharing usage in section 3.4 (p. 12)

Ferreira et al. (2022) : added citation related to travel behavior change in section 3.5 (p. 16)

Hensher et al. (2023) and Hensher et al. (2022) : added citation related to working from home in section 3.5 (p. 17)

Hintermann et al. (2023) : added citation related to the reduction of travel distance in section 3.5 (p. 17)

Huang et al. (2023), Loo and Huang (2022) : added text regarding working from home in section 3.5 (p. 17)

Javadinasr et al. (2022) : added citation (p. 16) and in Table 4 (p. 15) regarding travel behavior change in section 3.5

Lee et al. (2023) : added sentence (p. 9) and in Table 2 (p. 8) regarding impact of COVID-19 on mobility in section 3.2

Liu and Yamamoto (2022) : added citation related to stay-at-home effect in section 4.1 (p. 23)

Llaguno-Munitxa and Bou-Zeid (2023) : added citation related to air quality in section 3.4 (p. 13)

Mussone and Changizi (2023) : added text regarding factors affecting transport mode choice in section 3.5 (p. 16)

Nikolaidou et al. (2023) : added citation related to factors affecting public transport ridership in section 3.3 (p. 12)

Oestreich et al. (2023) : added citation related to travel behavior change in section 3.5 (p. 16)

Pan and He (2022) : added citation related to heterogenous impact of socioeconomic characteristics in section 3.2 (p. 9)

Pang et al. (2023) : added text (p. 5) and in Table 1 (p. 6) regarding relationship between high-speed railway and COVID-19 in section 3.1

Peng et al. (2023) : added citation related to agent-based model in section 3.2 (p. 9)

Rosik et al. (2022) : added citation related to government measures in section 4 (p. 18)

Sung (2022) : added sentence regarding social distancing effect in section 4.1 (p. 23)
