# Supplementary material for: Systematic literature review on impacts of COVID-19 pandemic and corresponding measures on mobility
Source: Transportation (Amst). 2023 Apr 25:1–55. Online ahead of print. doi: 10.1007/s11116-023-10392-2 (PMC10126540; doi:10.1007/s11116-023-10392-2)
Supplement: Supplementary file 1 — Supplementary Material 1 [file 11116_2023_10392_MOESM1_ESM.docx]

April 4, 2022

Prof. Dr. Kay W. Axhausen

Editor-in-Chief

*Transportation*

Dear Editor:

I wish to submit an original research article for publication in *Transportation*, titled “Systematic Literature Review on Impacts of COVID-19 Pandemic and Corresponding Measures on Mobility.” The paper was coauthored by Jin Ki Eom.

This study conducted a systematic review of the current literature on transport’s role in the COVID-19 response. We believe that our study makes a significant contribution to the literature because policy makers and transport planners need to understand and predict travel behaviors to facilitate managing people’s mobility during pandemics and other widespread crises. This comprehensive review provides valuable insights to facilitate better preparation for future health emergencies that affect urban transportation.

Further, we believe that this paper will be of interest to the readership of your journal because the findings have important theoretical and practical implications for transport planners and policymakers as well as future research in this field.

This manuscript has not been published or presented elsewhere in part or in entirety and is not under consideration by another journal. We have read and understood your journal’s policies, and we believe that neither the manuscript nor the study violates any of these. There are no conflicts of interest to declare.

Thank you for your consideration. I look forward to hearing from you.

Sincerely,

Kwang-Sub Lee, Ph.D.

Senior Researcher

Korea Railroad Research Institute

Railroad Policy Research Department

176 Railroad Museum Road

Uiwang-Si, Gyeonggi-Do, 16105, Korea

Phone: +82 31 460 5686

E-Mail: leeks33@krri.re.kr
